# ARFU-IDS: Robust Federated Unlearning for Transformer-Based Intrusion Detection in IoT and Sensor Networks

**DOI:** 10.3390/s26185911

**Published:** 2026-09-18

**Authors:** Xudong Yang, Zhenyu Zhang, Qiuyan Li, Zhenzhou Jing, Xuyao Lu, Yuxin Zhang, Junwen Chen

**Affiliations:** 1 School of Computer Science, Xi’an Polytechnic University, Xi’an 710048, China; 240721054@stu.xpu.edu.cn (Z.Z.); 240721043@stu.xpu.edu.cn (Z.J.); 250712139@stu.xpu.edu.cn (X.L.); 42309210822@stu.xpu.edu.cn (Y.Z.); 42309210908@stu.xpu.edu.cn (J.C.); 2 State Grid Henan Electric Power Company Economic and Technological Research Institute, Zhengzhou 450052, China; 250711010@stu.xpu.edu.cn

**Keywords:** wireless sensor networks, IoT security, federated learning, federated unlearning, transformer-based intrusion detection, attention-head attribution, adversarial robustness, execution integrity, class imbalance, edge intelligence

## Abstract

Federated intrusion detection systems (FL-IDSs) for IoT and wireless sensor networks (WSNs) must remove client contributions after training when consent changes, devices retire, or compromised nodes are identified. Existing federated unlearning methods are not well aligned with transformer-based IDSs because they use coarse attribution, provide limited trajectory verification, and handle concurrent deletion requests weakly under rare-attack imbalance conditions. This paper proposes ARFU-IDS, a robust federated unlearning framework for transformer-based IoT and sensor-network IDSs. ARFU-IDS combines attention-head attribution, dual-path layer criticality probing, manipulation-resistant iterative verification, and conflict-graph scheduling to remove target-client influence while preserving retained detection utility. Experiments on UNSW-NB15, CICIoT2023, and IoTID20 evaluated detection utility, rare-category recall, adversarial robustness with network-flow feature triggers, and concurrent unlearning. On UNSW-NB15, ARFU-IDS achieves 87.1% Macro-F1 and 77.6% rare-category recall, within 0.2 percentage points of full retraining. Under flow-feature trigger attacks, it reduces attack success rate to 8.2% at f=0.1 and 9.7% at f=0.2. For three concurrent deletion requests, it shortens online unlearning latency by 43.3% relative to sequential processing. These results show that ARFU-IDS offers a practical route to robust and efficient federated unlearning for transformer-based IDSs in IoT and sensor-network deployments.

## 1. Introduction

The Internet of Things (IoT), wireless sensor networks (WSNs), and edge intelligence are increasingly deployed in smart cities, Industrial IoT (IIoT), connected healthcare, intelligent transportation, and smart grids. These infrastructures continuously collect environmental states, device-operation traces, and network-flow records to support automated monitoring and decision making. At the same time, their large-scale, open-access patterns, heterogeneous protocols, and resource-constrained nodes expose them to a wide spectrum of attacks, including scanning, denial-of-service, botnet control, false-data injection, poisoning, and backdoor implantation [1,2,3,4]. Consequently, accurate, efficient, and distributed intrusion detection systems (IDSs) have become essential for securing IoT and sensor-network infrastructures [5].

An IDS identifies potential attacks by analyzing network traffic, communication behavior, or device-operation features. Conventional IDS pipelines often rely on centralized data collection and centralized model training, which can increase privacy exposure, communication burden, and cross-domain compliance risks. Federated learning (FL) offers a more suitable paradigm for privacy-sensitive IoT environments: clients keep raw traffic locally and collaboratively train a global IDS by exchanging model updates [3,4,6]. Existing FL-based IDS studies have mainly improved collaborative detection accuracy, non-IID robustness, class-imbalance handling, communication efficiency, and training-stage defense against malicious updates [1,2,7,8]. However, these works primarily focus on model training and aggregation. They do not sufficiently answer a later-stage maintenance question: after a global IDS has already absorbed a client’s historical contribution, how can that contribution be withdrawn without rebuilding the entire model?

This question is important in long-lived FL-IDS deployments. A client may need to withdraw its contribution because of privacy regulation, user consent revocation, data-governance policy, device retirement, or service termination. A node may also be identified after training as compromised, stale, poisoned, or adversarial, in which case its already-learned influence should be removed from the global detector. Full retraining after deleting the target client is a reliable baseline, but it is expensive in computation, communication, and response time, making it unsuitable for resource-constrained IoT and WSN environments. Federated unlearning (FU) therefore seeks to remove the influence of specified clients from a trained federated model while approximating the model that would have been obtained had those clients never participated [9,10,11,12].

Directly applying existing FU methods to transformer-based FL-IDSs remains insufficient. First, intrusion-detection datasets are severely imbalanced: rare but high-risk attack categories may contain only limited samples, so an unlearning update that removes a target client may also damage rare-category recognition. Second, transformer-based IDS models distribute discriminative attack information across multiple attention heads and projection subspaces; coarse neuron-, channel-, or layer-level updates may not separate target-client influence from task-critical rare-attack representations [13,14,15]. Third, the unlearning process itself may be manipulated by adversarial clients. If verification only checks the final model, abnormal intermediate update trajectories or delayed backdoor activation can remain undetected [16,17]. Fourth, in large-scale IoT systems, multiple clients may request deletion simultaneously. Sequential unlearning increases latency, while naive parallel unlearning may introduce conflicting updates and degrade detection utility. A practical FL-IDS unlearning method must therefore jointly support target-client removal, rare-attack preservation, execution integrity, and efficient multi-request scheduling.

This paper proposes ARFU-IDS, a robust federated unlearning framework tailored to transformer-based intrusion detection in IoT and sensor networks. The central research question is as follows: how can a transformer-based FL-IDS remove target-client contributions without full retraining while preserving rare-attack detection, resisting adversarial manipulation, and processing concurrent deletion requests efficiently? ARFU-IDS answers this question through four coordinated modules. The Attention-Head Attribution Graph (AHAG) localizes removal-sensitive and rare-category-critical attention heads through QKV Frobenius-gradient scoring. Dual-Path Layer Criticality Probing (DPLCP) distinguishes task-critical layers from adversary-suspected layers to avoid protecting malicious representations. Manipulation-Resistant Iterative Verification with Audit (MRIVA) monitors the unlearning trajectory round by round and performs post hoc audits. Finally, a conflict-graph scheduler coordinates multiple deletion requests according to their overlap in critical representation space.

The main contributions of this paper are summarized as follows:

- We formulate client-contribution removal for transformer-based FL-IDS and jointly consider rare-attack preservation, adversarially manipulated unlearning, and concurrent deletion requests in one framework.
- We propose the AHAG and DPLCP to perform attention-head-level attribution and dual-path layer analysis, reducing the risk that unlearning removes target-client influence at the cost of damaging rare-category detection.
- We design MRIVA and a conflict-aware scheduler to improve execution integrity and multi-client unlearning efficiency under adversarial and class-imbalanced conditions.
- We evaluate ARFU-IDS on UNSW-NB15, CICIoT2023, and IoTID20 from the perspectives of detection utility, rare-category recall, unlearning fidelity, adversarial robustness, concurrent processing efficiency, ablation, and parameter sensitivity.

The remainder of this paper is organized as follows. Section 2 reviews FL-based IDSs, federated unlearning, adversarial unlearning security, and transformer-oriented IDSs and positions ARFU-IDS against existing work. Section 3 presents the system model, threat model, backbone architecture, and the four modules of ARFU-IDS. Section 4 reports the experimental results in the same order as the design requirements. Section 5 discusses limitations and deployment implications, Section 6 concludes the paper, and Appendix A provides per-seed raw results.

## 2. Related Work and Research Gaps

### 2.1. FL-Based Intrusion Detection in IoT and Sensor Networks

The first line of research relevant to ARFU-IDS concerns intrusion detection for IoT, WSN, and edge environments. Early IDS studies emphasized centralized traffic collection, signature matching, or classical machine-learning pipelines, but the sensitivity and scale of IoT traffic make centralized training increasingly difficult. Recent surveys and system studies show that FL has become a natural solution for distributed IDSs because it reduces raw-data sharing while allowing multiple organizations, gateways, or edge nodes to jointly train detection models [1,2,3,4]. In parallel, semantic and self-supervised traffic representations improve the modeling of complex request bodies, URLs, and contextual features in IoT and sensor-network IDSs [5]. Transformer-based architectures further extend this direction by using multi-head self-attention to capture feature interactions in tabular or flow-level traffic [13,14]. Security-oriented studies also show that FL-based IDSs must withstand label flipping, poisoning, and other adversarial updates during training [7,8]. Together, these works establish the feasibility of privacy-preserving IDS training, but their main focus remains on training-stage detection accuracy, robustness, and communication efficiency. They rarely study what happens after deployment when a particular client’s historical contribution must be removed from the already-trained IDS.

### 2.2. Federated Unlearning and Client Contribution Removal

The second line of work studies machine unlearning and federated unlearning. General FU surveys summarize three major families of methods: retraining-based removal, historical-update reconstruction, and approximate parameter correction [9]. Projected Gradient Ascent (PGA) performs client removal through constrained reverse updates [10], while Fast-FedUL/FedOSD-type approaches reduce cost by avoiding extensive retraining with skewed data [11]. More recent methods explore sparse adapters and selective parameter updates, such as FUSED [12] and USP [18]. These works provide important foundations for efficient client removal, but most of them evaluate general classification models or non-transformer backbones. They do not explicitly protect rare IDS categories, do not analyze transformer attention-head structure, and usually treat each unlearning request in isolation.

### 2.3. Adversarial, Transformer-Oriented, and Concurrent Unlearning Security

The third line of work shows why FU for security-critical IDSs must be adversary-aware. FedSweep demonstrates that backdoor representations can be activated or exposed through unlearning requests, and BadUnlearn shows that adversarial per-round updates can corrupt the unlearning process itself [16,17]. These findings are consistent with broader observations that the FL-based IDS is vulnerable to label-flipping, poisoning, and adaptive adversarial updates [7,8]. At the same time, transformer interpretability research indicates that attention-head and projection-level structures provide more meaningful attribution units than coarse neuron-level scores for transformer models [15]. Recent transformer-based IDS studies confirm the practical relevance of this architecture in IoT detection tasks [5,13,14]. However, adversarial FU defenses such as FedSweep and UnlearnGuard focus mainly on backdoor or update filtering, while practical FU methods reviewed above mainly prioritize efficient client removal and do not include rare-category preservation or trajectory-level IDS auditing [12,16,17,18]. Hence, existing studies address isolated pieces of the problem but do not yet provide a transformer-oriented, adversary-aware, and conflict-aware FU framework for the IoT IDS.

### 2.4. Research Positioning and Gap Analysis

The above literature forms a layered progression: the FL-based IDS improves privacy-preserving collaborative detection; FU methods enable post-training client contribution removal; adversarial FU research reveals that the unlearning process itself can be exploited; and a transformer-based IDS motivates finer attribution units than neurons or channels. ARFU-IDS is positioned at the intersection of these four directions. Compared with the standard FL-IDS, it focuses on post-training removal rather than only collaborative training. Compared with generic FU, it explicitly protects rare-attack categories and analyzes transformer attention heads. Compared with adversarial FU defenses, it verifies the unlearning trajectory instead of relying only on a final-state audit. Compared with concurrent FU strategies, it adds conflict-aware scheduling under IDS rare-category constraints. This positioning motivates the design of a transformer-oriented, adversary-aware, and conflict-aware federated unlearning framework tailored for IoT intrusion detection.

Table 1 maps representative methods against the four design requirements: P1, transformer head-level attribution; P2, execution-trajectory integrity; P3, adversary-aware layer separation; and P4, concurrent fair scheduling.

ARFU-IDS is designed to jointly address these four gaps in the transformer-based IoT IDS setting: target-client removal, rare-category preservation, adversary-aware trajectory verification, and concurrent-request scheduling. This gap-to-module mapping also determines the organization of Section 3: AHAG addresses P1, MRIVA addresses P2, DPLCP addresses P3, and the conflict-graph scheduler addresses P4. The mapping is derived from the literature reviewed above and from the four design requirements stated in the Introduction.

## 3. Materials and Methods

This section follows the problem-to-method progression established in Section 2.4. We first define the system and threat model, then introduce the backbone architecture and framework overview, formalize notation, describe the four modules corresponding to P1–P4, and finally specify the experimental setup used to evaluate each design requirement.

### 3.1. System and Threat Model

#### 3.1.1. Standard Setting

We consider a standard client-level federated unlearning setting with *K* clients holding local flow-level IDS datasets $D_{k}$. A global model $\theta^{*}$ is trained over $T_{\mathrm{train}}$ rounds of FedAvg [6]. Target clients $K_{u}$ submit requests to remove their contributions $D_{u}$ from the model while preserving the retained utility. The server may hold a retained validation set $D_{\mathrm{val}}$, a held-out partition of $D\setminus D_{u}$, used only for aggregate validation losses, AHAG/DPLCP scoring, and MRIVA reference-gradient estimation; it is not used to retrain on target-client records.

#### 3.1.2. Assumptions

The following assumptions define the scope of this work:

A1. The server and non-target clients are honest.
A2. At most a fraction *f* of the $|K_{u}|$ unlearning clients are malicious. Malicious clients submit adversarially crafted per-round gradient updates consistent with the BadUnlearn model [17].
A3. The server-held validation set $D_{\mathrm{val}}$ is not compromised by the adversary.
A4. The retained validation set has declared support for the retained rare categories used in the AHAG. If deployment rules prohibit any server-held validation set, or if rare-category support is too small or severely skewed, ARFU-IDS is treated as operating outside its standard assumption and must use the fallback policy below rather than claiming full rare-category preservation.

#### 3.1.3. Validation-Set Support and Fallback Policy

The role of $D_{\mathrm{val}}$ is limited to server-side estimation of validation losses, AHAG Frobenius-gradient scores, DPLCP layer ablation, and MRIVA reference-gradient statistics. ARFU-IDS is therefore not a zero-knowledge FL protocol: if the server has no retained validation evidence, its head attribution and trajectory verification become under-determined. When $D_{\mathrm{val}}$ is tiny or skewed, the framework records the per-class support $n_{c}^{\mathrm{val}}$ for every $c\in C_{\mathrm{rare}}$ and uses bootstrap resampling over $D_{\mathrm{val}}$ to estimate the uncertainty of AHAG and DPLCP scores. If a rare category does not meet the minimum validation-support requirement used by the deployment, the implementation enters a conservative mode: the unsupported category is reported as outside the rare-preservation claim, MRIVA thresholds are not relaxed, concurrent requests involving that category are serialized, and a failed final audit triggers full retraining or manual review rather than deployment. This policy clarifies what happens under weak retained-validation conditions and avoids overstating ARFU-IDS in true zero-knowledge FL environments.

#### 3.1.4. Extended Conditions

Two conditions capture the adversarial and multi-tenant dimensions of this setting.

**Condition C1 (Execution integrity).** The gradient trajectory ${\left\{\Delta \theta^{\left(t\right)}\right\}}_{t=1}^{T_{\mathrm{unl}}}$ must be consistent with honest gradient sculpting for the losses defined by the AHAG and DPLCP, verified by MRIVA with false-alert rate $\leq \eta_{\mathrm{FA}}$.

**Condition C2 (Concurrent fairness).** When $|K_{u}|>1$, the per-client ${\mathrm{Recall}}_{\mathrm{rare}}^{\left(k\right)}$ after concurrent unlearning must satisfy ${\mathrm{Recall}}_{\mathrm{rare}}^{\left(k\right)}\geq 0.9\cdot {\mathrm{Recall}}_{\mathrm{rare}}^{\left(k\right)}\left(\theta_{\mathrm{retrain}}\right)$ for all retained rare categories.

### 3.2. Backbone Architecture

ARFU-IDS is evaluated on a Tab Transformer backbone [13] with $L_{T}$ transformer layers, *H* attention heads per layer, and hidden dimension $d_{\mathrm{model}}$. The Tab Transformer processes each network flow as a sequence of *M* feature tokens. We use this backbone because multi-head self-attention provides explicit head-level subspaces, enabling the AHAG to localize the parts of the model that jointly encode target-client influence and rare-category evidence.

### 3.3. Framework Overview

**Figure 1:**
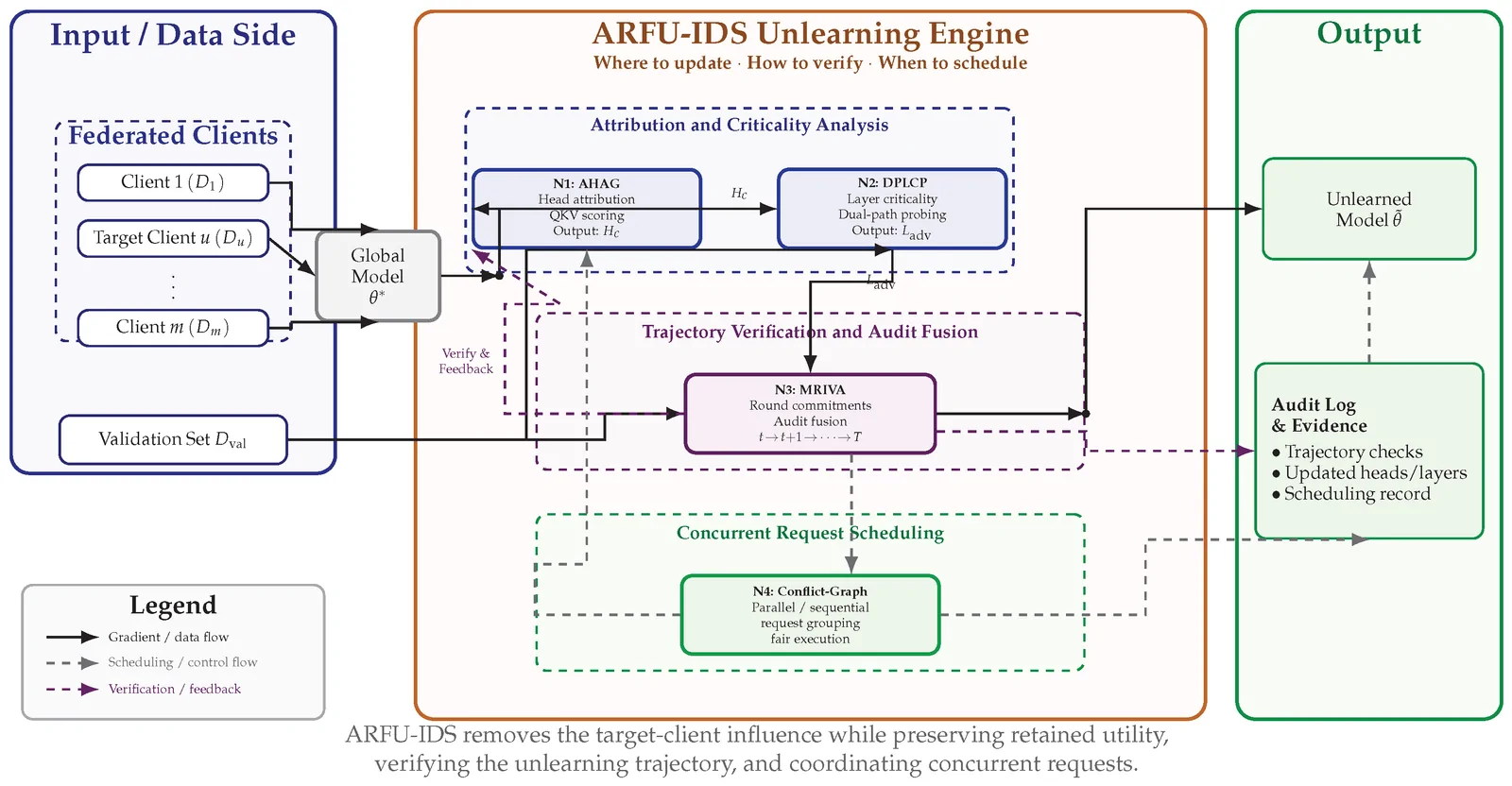
Overall federated unlearning pipeline of ARFU-IDS. The four modules jointly determine *what* to update (AHAG, DPLCP), *whether* the update trajectory is safe (MRIVA), and *how* concurrent requests are executed (conflict-graph scheduler), producing the unlearned model $\tilde{\theta }$ and a complete audit log.

Figure 1 illustrates the overall ARFU-IDS pipeline. Representative attention-head attribution heatmaps, embedding projections, and per-seed training/validation loss convergence curves are provided in Supplementary Figures S1–S3. AHAG and DPLCP determine *where* gradients are applied (which heads and which layers). MRIVA monitors *how* the gradient trajectory evolves across rounds. The conflict-graph scheduler decides *when* concurrent requests can be processed in parallel versus sequentially. Together, the four modules produce the unlearned model $\tilde{\theta }$ and a complete audit log.

Table 2 and Table 3 translate the unresolved limitations identified in Section 2.4 into a gap-to-module map for ARFU-IDS.

### 3.4. Notation

Table 4 summarizes the main symbols used throughout the methodology.

### 3.5. Module N1: AHAG—Attention-Head Attribution Graph

#### 3.5.1. Motivation

In transformer models, each attention head h∈{1,…,H} in layer *l* encodes a distinct contextual aggregation pattern. Rare-attack categories (e.g., Worms and Shellcode) are distinguished by subtle long-range dependencies captured by specific heads. Uniform gradient ascent destroys rare-category detection at the same rate as target-client erasure, causing the rare-category recall collapse observed in standard FU applied to transformers.

#### 3.5.2. Head-Level Attribution

We attribute over the full head projection—query ($W_{h}^{Q}\in R^{d_{k}\times d_{\mathrm{model}}}$), key ($W_{h}^{K}$), and value ($W_{h}^{V}\in R^{d_{v}\times d_{\mathrm{model}}}$)—by computing the Frobenius norm of each projection matrix’s gradient with respect to the class log-probability. Inspired by gradient-weighted attention rollout [15] but adapted to use Frobenius-norm gradient aggregation only, we compute a dimensionally consistent scalar per head:

(1) $$\begin{array}{cc} s_{h}^{QK}\left(x,c\right) & ={∥\nabla_{W_{h}^{Q}}\log p_{\theta^{*}}\left(c|x\right)∥}_{F}+{∥\nabla_{W_{h}^{K}}\log p_{\theta^{*}}\left(c|x\right)∥}_{F},\\ s_{h}^{V}\left(x,c\right) & ={∥\nabla_{W_{h}^{V}}\log p_{\theta^{*}}\left(c|x\right)∥}_{F},\\ A(h_{l},\;c) & =E_{\left(x,y=c\right)∼D_{\mathrm{val}}}{\left[\left(s_{h}^{QK}\left(x,c\right)+s_{h}^{V}\left(x,c\right)\right)\right]}^{+}, \end{array}$$

where ${\left[z\right]}^{+}=\max\left(z,0\right)$ denotes positive-part clamping.

#### 3.5.3. Rationale for Frobenius-Norm Gradient Scoring

The Frobenius norm captures the total *magnitude* of the gradient across the entire projection matrix, making it sensitive to perturbations that affect many neurons simultaneously—exactly the pattern expected when a head encodes a rare category. Per-neuron $\ell_{2}$ norms would require an additional aggregation step and introduce arbitrary choices (e.g., sum vs. max); the Frobenius norm naturally collapses the matrix gradient to a single scalar and is orthogonally invariant, giving a dimensionally consistent matrix-level sensitivity score for each head.

#### 3.5.4. Conflict Head Identification

The *pseudo-category label* $c_{u}$ is a centroid-derived target-client attribution label, not a newly introduced ground-truth attack class or a relabeling of traffic records. It is defined as $c_{u}=\arg\max_{c}\;p_{\theta^{*}}\left(c|{\bar{x}}_{u}\right)$, where ${\bar{x}}_{u}$ is the per-client centroid of training embeddings on $D_{u}$. In practice, $c_{u}$ is computed once before the first unlearning round from the client’s locally held data, and the server uses it solely to compute AHAG conflict scores; no raw data leaves the client and all original traffic labels remain unchanged. When the target client contains data from multiple categories, $c_{u}$ identifies the model-predicted category most associated with the target client’s embedding centroid, consistent with the single-request removal framing of this work. Multi-category unlearning is an open direction noted in Section 6.

Let $C_{\mathrm{rare}}$ denote the fixed set of semantically defined, under-represented high-risk anomalous categories specified in Section 3.10; the set is read from class_mappings.json and kept identical across all seeds before Dirichlet partitioning. The conflict score for head $h_{l}$ is

$$\mathrm{Conflict}\left(h_{l}\right)=A\left(h_{l},\;c_{u}\right)\cdot \max_{c\in C_{\mathrm{rare}}}A\left(h_{l},\;c\right).$$

Heads above the 75th-percentile conflict threshold form $H_{\mathrm{conflict}}\equiv H_{c}$. Sensitivity to this threshold is analyzed in Section 4.4.

#### 3.5.5. Differentiated Head Gradient Scaling

Let σ(·) denote the sigmoid function and τ the 75th-percentile conflict value. The per-head learning rate is

(2) $$\alpha \left(h_{l}\right)=\alpha_{\mathrm{base}}\cdot \left(1-\lambda_{c}\cdot \sigma \left(\mathrm{Conflict}\left(h_{l}\right)-\tau \right)\right),$$

where $\lambda_{c}=0.8$. High-conflict heads receive approximately 20% of $\alpha_{\mathrm{base}}$; non-conflict heads receive the full rate, preserving rare-category attention patterns during unlearning.

**Figure 2:**
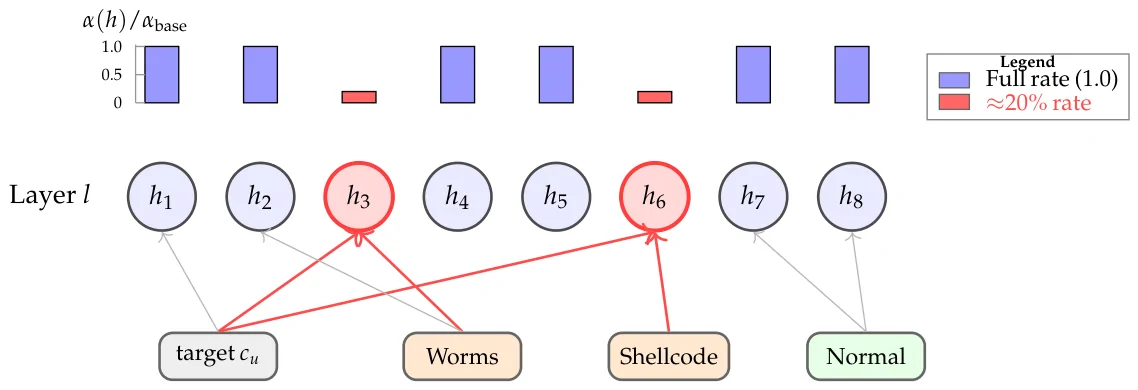
AHAG head attribution and differentiated gradient scaling. Conflict heads (red) receive attenuated updates; blue denotes non-conflict heads and base-rate updates, orange denotes rare categories, green denotes the normal category, and gray denotes the target category or non-conflict attribution links.

Figure 2 illustrates how the AHAG isolates the small fraction of heads jointly attributable to the target client and a rare category.

### 3.6. Module N2: DPLCP—Dual-Path Layer Criticality Probing

#### 3.6.1. Dual-Signal Design

Standard LCP ablates each layer *l* and measures Macro-F1 drop $\Delta_{l}^{\mathrm{task}}$ on $D_{\mathrm{val}}$. This score can be inflated when a layer encodes a backdoor pattern [16]. DPLCP adds a second, orthogonal signal—gradient consistency discrepancy (GCD)—and assigns divergent update policies based on both jointly.

#### 3.6.2. Task-Path Criticality

Following standard layer-wise criticality probing, for each layer *l*, $\Delta_{l}^{\mathrm{task}}$ is computed by ablation on $D_{\mathrm{val}}$. Layers with $\Delta_{l}^{\mathrm{task}}\geq \delta_{\mathrm{crit}}=0.05$ (a 5 pp Macro-F1 drop) form the task-critical set $L_{\mathrm{task}}$. This threshold is fixed throughout all experiments as a conservative criterion separating important layers from noise-level fluctuations. The novelty of DPLCP lies not in this signal but in pairing it with the adversary-aware signal below.

#### 3.6.3. Adversary-Suspected Path Analysis

For each layer *l*:

$${\mathrm{GCD}}_{l}=1-\cos\;\left(\nabla_{l}L_{\mathrm{forget}}\left(\theta^{*};\;D_{u}\right),\;\nabla_{l}L_{\mathrm{preserve}}\left(\theta^{*};\;D_{\mathrm{val}}\right)\right),$$

where $L_{\mathrm{forget}}\left(\theta^{*};D_{u}\right)=-\frac{1}{|D_{u}|}\sum_{\left(x,y\right)\in D_{u}}\log p_{\theta^{*}}\left(y|x\right)$ is the cross-entropy loss on $D_{u}$. Gradient ascent on this loss increases prediction error on $D_{u}$, pushing representations away from the target client’s distribution. A high ${\mathrm{GCD}}_{l}$ indicates strong misalignment between forgetting and preservation gradients—a signature of adversarial representations [16]. Layers with ${\mathrm{GCD}}_{l}\geq \delta_{\mathrm{adv}}=0.7$ form the adversary-suspected set $L_{\mathrm{adv}}$. The value $\delta_{\mathrm{adv}}=0.7$ is selected by grid search over {0.5,0.6,0.7,0.8,0.9} on a held-out portion of the UNSW-NB15 validation set for f=0.1, minimizing ASR while keeping Macro-F1 within 1.5 pp of the full-system baseline. Sensitivity to $\delta_{\mathrm{adv}}$ is reported in Section 4.4. We acknowledge that gradient-based adversary detection shares conceptual roots with federated attacks studied by Jebreel et al. [7] and Shahid et al. [8]; the key distinction is that DPLCP operates on the *unlearning* gradient trajectory rather than the training gradient, and the GCD signal is computed layer-wise rather than update-wise, providing finer-grained separation of poisoned and task-critical representations.

#### 3.6.4. Divergent Update Policy


(3) $$\mathrm{Update}\left(l\right)=\left\{\begin{array}{cc} \mathrm{Fisher}\text{-}\mathrm{EWC}\;\left(\mathrm{protective}\right)\; & l\in L_{\mathrm{task}}\setminus L_{\mathrm{adv}},\\ \mathrm{Aggressive}\;\mathrm{overwriting}\; & l\in L_{\mathrm{adv}},\\ \mathrm{Standard}\;\mathrm{gradient}\;\mathrm{ascent}\; & \mathrm{otherwise}. \end{array}\right.$$

**Figure 3:**
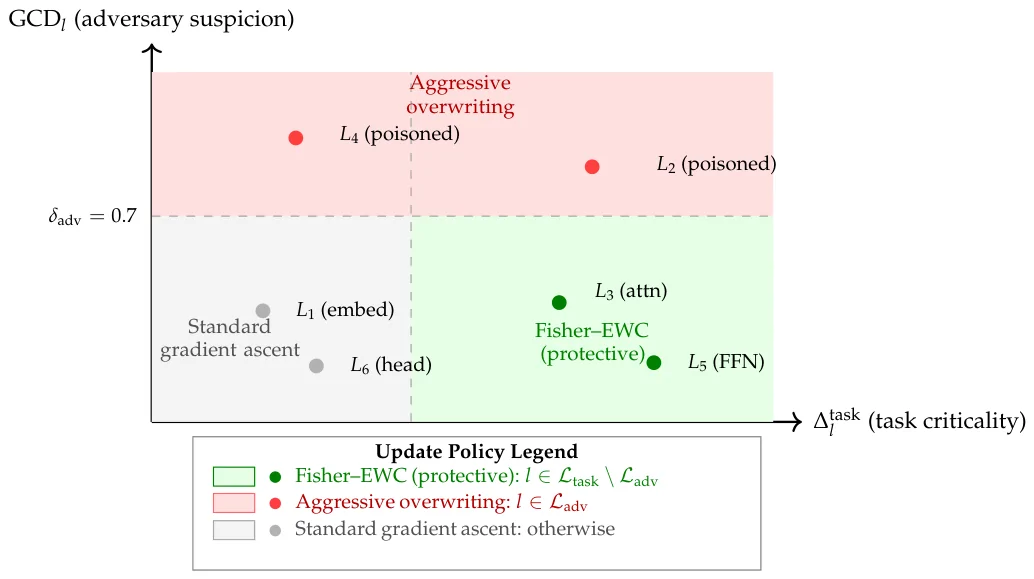
DPLCP dual-signal classifier. Each layer is positioned by task criticality $\Delta_{l}^{\mathrm{task}}$ and gradient consistency discrepancy ${\mathrm{GCD}}_{l}$, and assigned to one of three update regions.

Aggressive overwriting applies $\theta_{l}\leftarrow \theta_{l}+\eta_{\mathrm{adv}}\nabla_{l}L_{\mathrm{forget}}$ with $\eta_{\mathrm{adv}}=5\alpha_{\mathrm{base}}$, overriding Fisher–EWC protection that would otherwise shield backdoor representations. Figure 3 visualizes the dual-signal classifier.

### 3.7. Module N3: MRIVA—Manipulation-Resistant Iterative Verification with Audit

#### 3.7.1. Design Rationale

The post hoc audit branch of MRIVA combines LiRA-based membership inference with Dempster–Shafer fusion to produce a post hoc decision $V^{\mathrm{post}}$. BadUnlearn [17] and FedSweep [16] have shown that any final-state audit can be defeated by trajectory manipulation that causes the final model to pass membership-inference testing while remaining poisoned on triggered inputs. MRIVA therefore adds a per-round trajectory branch that audits the *process* rather than only the *outcome*; both branches are fused in Equation (4).

#### 3.7.2. Round-by-Round Commitment

At each unlearning round $t\in \{1,\ldots ,T_{\mathrm{unl}}\}$:

$$c_{t}=\mathrm{Hash}\;\left(\Pi_{H_{c}\cup L_{\mathrm{adv}}}\Delta \theta^{\left(t\right)}\right),$$

where $\Pi_{H_{c}\cup L_{\mathrm{adv}}}$ projects onto the AHAG conflict heads and DPLCP adversary-suspected layers.

#### 3.7.3. Robustness of the Reference Gradient Estimate

The reference direction ${\hat{g}}_{t}$ is estimated from $D_{\mathrm{val}}$ over $R_{\mathrm{ref}}=10$ honest reference trials. Let $\mu_{t}$ and $\sigma_{t}$ denote the empirical mean and standard deviation of ${∥\Delta \theta_{H_{c}}^{\left(t\right)}-{\hat{g}}_{t}∥}_{2}$ over those trials. The round-adaptive tolerance $\rho_{t}=\mu_{t}+3\sigma_{t}$ ensures a per-round false-alarm probability $\eta_{\mathrm{FA}}^{\left(1\right)}\approx 0.003$ in the Gaussian noise model, giving a per-session bound of ≤0.048 for $T_{\mathrm{unl}}=20$ and f≤0.2 (see Proposition 1). Because $H_{c}$ is derived from the pre-unlearning model using server-controlled $D_{\mathrm{val}}$, corrupting it would require compromising the server’s validation set, which lies outside the stated threat model.

#### 3.7.4. Per-Round Verification

At the end of each round *t*

$$V_{t}=\left\{\begin{array}{cc} \mathrm{PASS} & {∥\Delta \theta_{H_{c}}^{\left(t\right)}-{\hat{g}}_{t}∥}_{2}\leq \rho_{t},\\ \mathrm{FAIL} & \mathrm{otherwise}. \end{array}\right.$$

#### 3.7.5. Integrated Deployment Decision


(4) $$V_{\mathrm{final}}=\left\{\begin{array}{cc} \mathrm{PASS} & V^{\mathrm{post}}=\mathrm{PASS}\;\wedge \;\sum_{t}1\left[V_{t}=\mathrm{FAIL}\right]\leq f\cdot T_{\mathrm{unl}},\\ \mathrm{FAIL} & \mathrm{otherwise}. \end{array}\right.$$

**Proposition** **1** (MRIVA Guarantee)**.** *In the stated threat model with at most f fraction of adversarial rounds and a collision-resistant hash function,*

*1.*  (False-alert bound) *The per-session probability that any honest round is incorrectly flagged FAIL is at most $\eta_{FA}\leq T_{unl}\left(1-f\right)\cdot \eta_{FA}^{\left(1\right)}$, where $\eta_{FA}^{\left(1\right)}\approx 0.003$ in the Gaussian noise model.*
*2.*  (Out-of-budget detection) *If the adversary uses more than $f\cdot T_{unl}$ rounds with deviation exceeding $\rho_{t}$, MRIVA issues $V_{final}=FAIL$ with probability 1.*
*3.*  (In-budget constraint) *An adversary respecting the declared f budget faces a trade-off: high-deviation rounds accumulate FAIL flags, while low-deviation rounds restrict the per-round backdoor signal to within the honest gradient variance. The residual ASR is empirically negligible, as evaluated later in Section 4.2.*

**Proof.**  *Part 1 (False-alert bound).* $\rho_{t}=\mu_{t}+3\sigma_{t}$ is the empirical 3σ upper quantile of honest-round deviations from $R_{\mathrm{ref}}$ reference trials, so each honest round satisfies $V_{t}=\mathrm{PASS}$ with probability ≥$1-\eta_{\mathrm{FA}}^{\left(1\right)}\approx 0.997$. By the union bound over at most $T_{\mathrm{unl}}\left(1-f\right)$ honest rounds, the per-session false-alert rate is at most $T_{\mathrm{unl}}\left(1-f\right)\cdot \eta_{\mathrm{FA}}^{\left(1\right)}\leq 0.048$ for $T_{\mathrm{unl}}=20$, f≤0.2.

*Part 2 (Out-of-budget detection).* By collision resistance, $c_{t}$ uniquely identifies $\Pi_{H_{c}\cup L_{\mathrm{adv}}}\Delta \theta^{\left(t\right)}$. Any round with deviation $>\rho_{t}$ is flagged FAIL with probability 1. $V_{\mathrm{final}}$ is PASS iff $\sum_{t}1\left[V_{t}=\mathrm{FAIL}\right]\leq f\cdot T_{\mathrm{unl}}$. If the adversary uses more than $f\cdot T_{\mathrm{unl}}$ high-deviation rounds, the FAIL count exceeds the threshold and $V_{\mathrm{final}}=\mathrm{FAIL}$ with probability 1.

*Part 3 (In-budget constraint).* An adversary respecting *f* may use at most $f\cdot T_{\mathrm{unl}}$ adversarial rounds. Each such round either deviates $>\rho_{t}$ (contributing a FAIL flag) or stays ≤$\rho_{t}$ (restricting the backdoor signal to within the honest gradient variance). The maximum achievable backdoor signal is therefore bounded; residual ASR is empirically negligible, as evaluated in Section 4.2.    □

**Figure 4:**
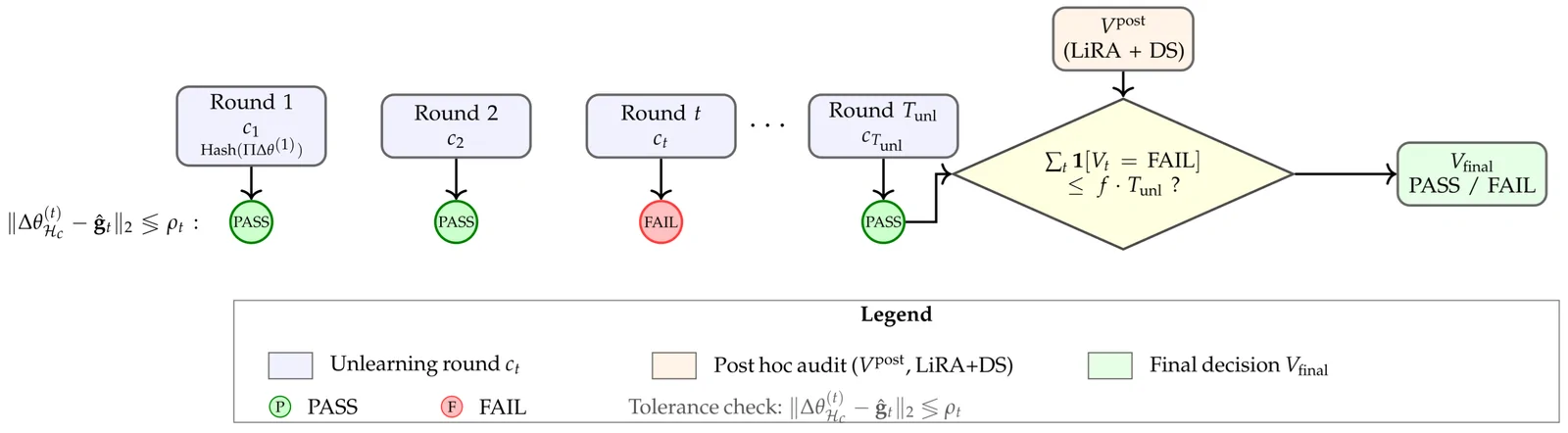
MRIVA per-round trajectory verification. Each round commits a hash $c_{t}$ of the projected update and is flagged when the deviation from the reference direction ${\hat{g}}_{t}$ exceeds the adaptive tolerance $\rho_{t}$. The accumulated evidence is combined with the post hoc audit $V^{\mathrm{post}}$ to produce the final deployment decision.

Figure 4 depicts the per-round verification flow.

### 3.8. Module N4: Conflict-Graph Multi-Client Scheduling

#### 3.8.1. Overlap Quantification

For concurrent requests $\left\{k_{u_{1}},\ldots ,k_{u_{m}}\right\}$, the inter-client conflict weight is the Jaccard similarity of conflict head sets:

$$w\left(k_{u_{i}},k_{u_{j}}\right)=\frac{|H_{\mathrm{conflict}}^{\left(i\right)}\cap H_{\mathrm{conflict}}^{\left(j\right)}|}{|H_{\mathrm{conflict}}^{\left(i\right)}\cup H_{\mathrm{conflict}}^{\left(j\right)}|}.$$

#### 3.8.2. Scheduling Policy

An undirected conflict graph $G_{c}$ is constructed with nodes $\left\{k_{u_{i}}\right\}$ and edges weighted by *w*. A conflict threshold $\tau_{c}=0.3$ separates low-conflict from high-conflict pairs. Unlearning proceeds in two phases:

1. *Parallel phase*: connected components with maximum edge weight ≤$\tau_{c}$ are processed simultaneously with independent AHAG+DPLCP gradient sculpting.
2. *Sequential phase*: within high-conflict components (maximum edge weight >$\tau_{c}$), clients are processed in descending order of $\sum_{j}w\left(k_{u_{i}},k_{u_{j}}\right)$ (highest conflict first), with the AHAG recomputed after each removal. Removing the most broadly conflicting client first shrinks the conflict sets of all remaining clients, reducing subsequent gradient interference.

**Figure 5:**
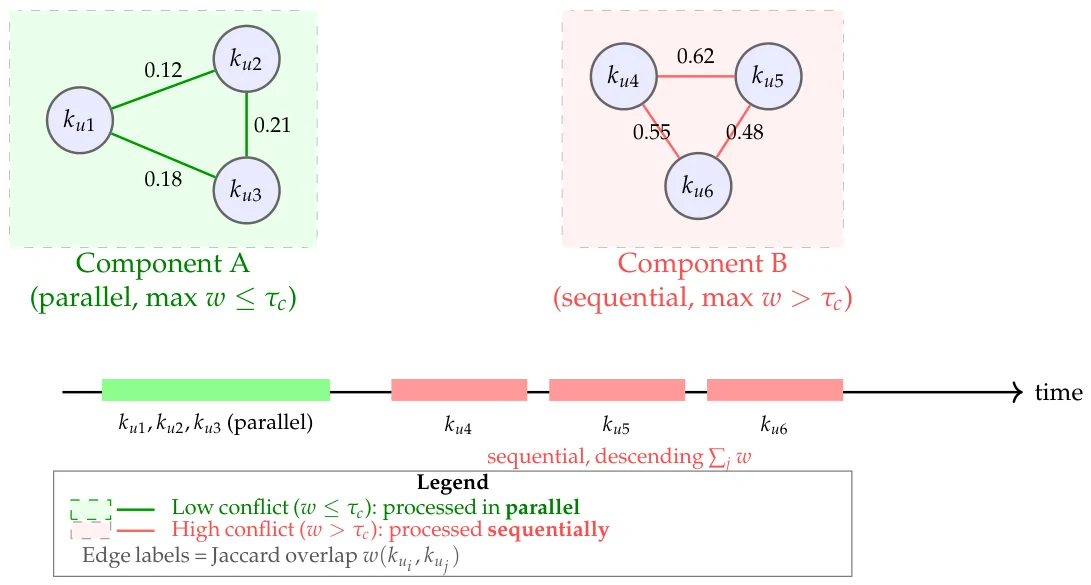
Conflict-graph scheduling for concurrent unlearning. Low-overlap components are processed in parallel; high-overlap components are serialized by descending conflict mass.

Figure 5 illustrates this policy for six concurrent requests.

### 3.9. Algorithmic Summary

Algorithms 1 and 2 provide a compact phase-based summary of the ARFU-IDS unlearning procedure, following the earlier single-procedure style while splitting the content only where needed for page layout.
**Algorithm 1:** ARFU-IDS attribution, criticality analysis, and scheduling
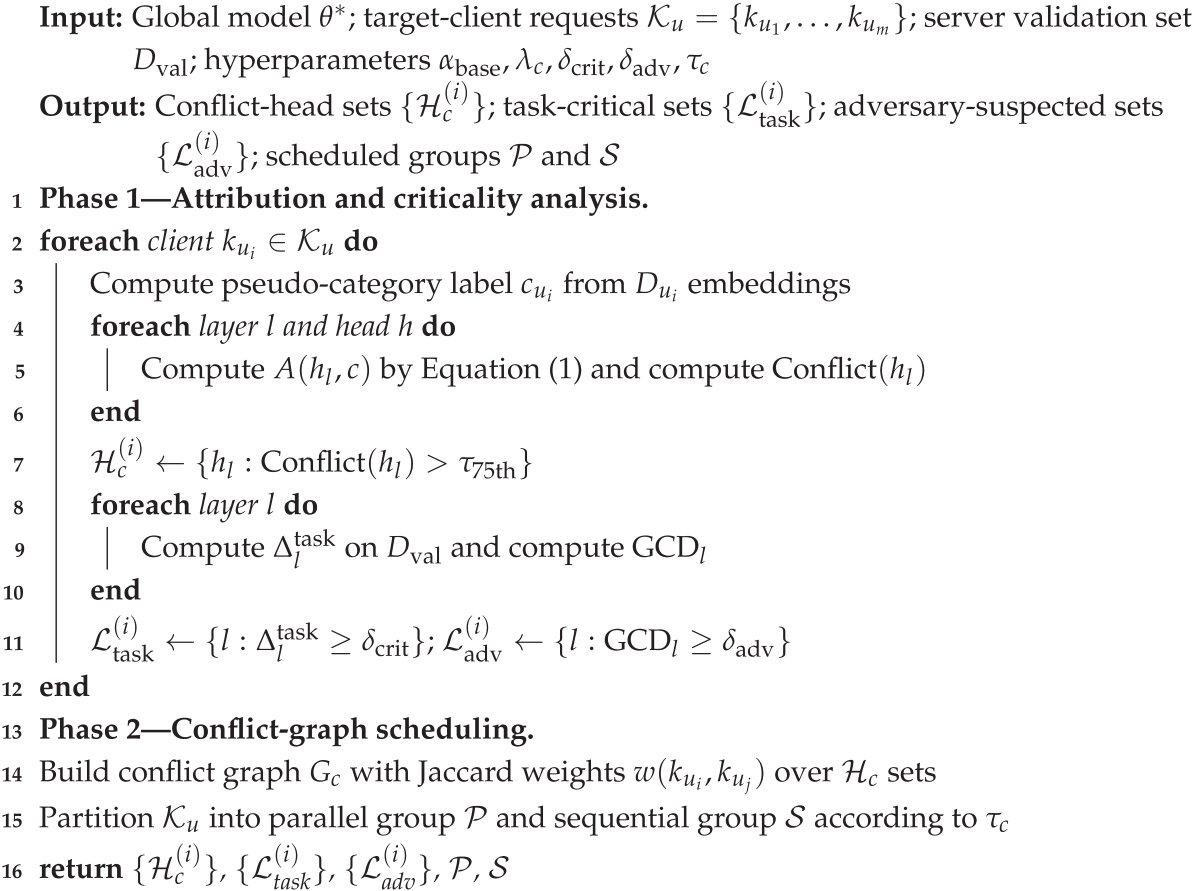

**Algorithm 2:** ARFU-IDS verified unlearning and final audit decision
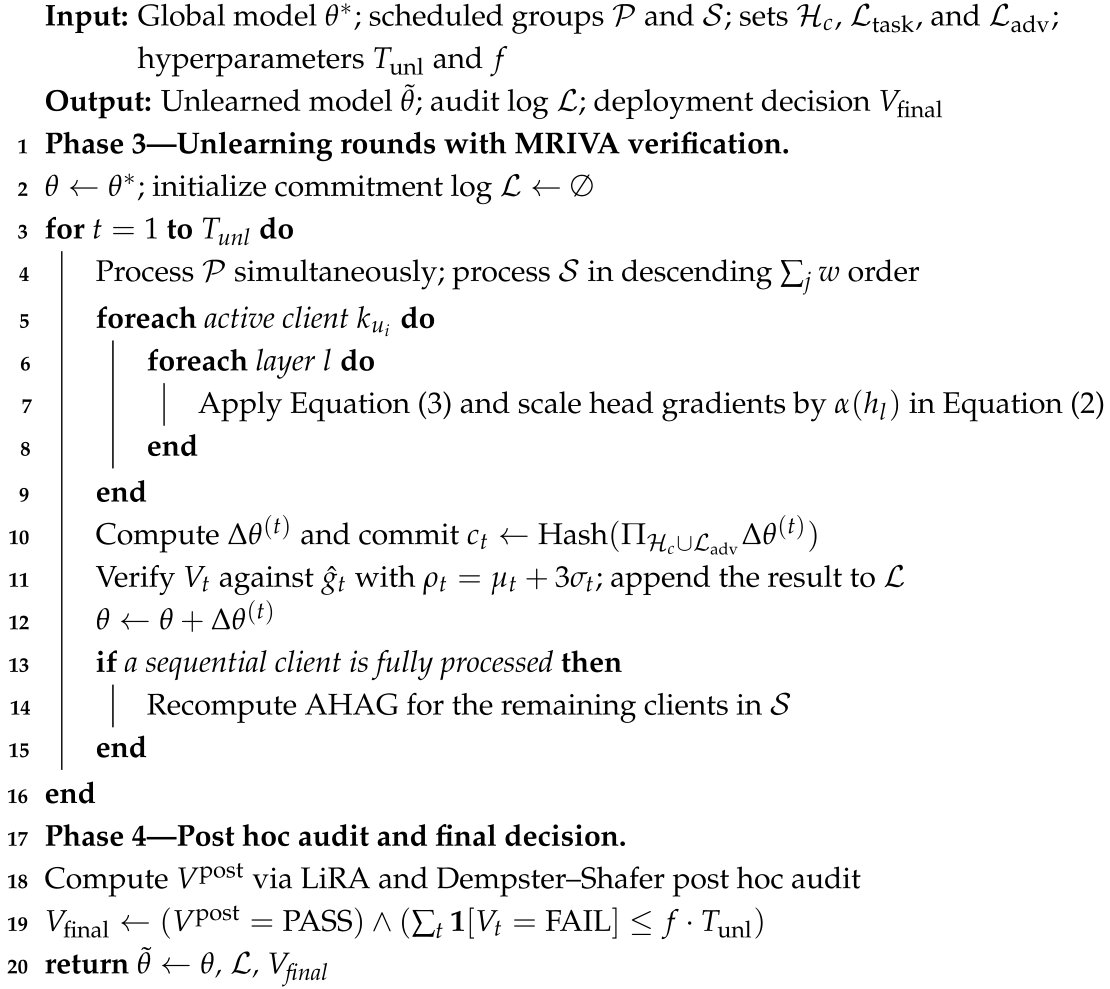


### 3.10. Experimental Setup

#### 3.10.1. Datasets

Three benchmark datasets were selected to cover distinct IoT/sensor traffic characteristics and class-imbalance levels.

- **UNSW-NB15** [19]: 2.54M records and 10 attack categories (Normal, Fuzzers, Analysis, Backdoors, DoS, Exploits, Generic, Reconnaissance, Shellcode, Worms), with class imbalance up to 5300:1. Its extreme rarity of Worm and Shellcode traffic makes it the primary benchmark for rare-category preservation.
- **CICIoT2023** [20]: 1.5 M IoT network flows, 34 categories encompassing DDoS, DoS, Mirai, and benign traffic. It reflects the traffic diversity of contemporary IoT deployments.
- **IoTID20** [21]: 625 K smart-home records, 6 categories (Normal, Mirai, DoS, Scan, MITM, Data Exfiltration), and 120:1 imbalance. Its controlled scale enables complementary validation.

These three datasets collectively span different attack spaces, imbalance levels, and traffic sources, making them representative of realistic IoT and WSN deployments. We acknowledge that the UGRansome benchmark offers an emerging alternative; its inclusion is deferred to future work.

#### 3.10.2. Data Preprocessing and Dataset Splits

All preprocessing steps were applied identically across all methods and seeds to ensure fair comparison.

- **Feature selection.** For UNSW-NB15, attack_cat was first used as the multiclass target label; we then used 42 numerical flow-level features after removing identifier columns and non-feature label fields (id, attack_cat string, label). For CICIoT2023 we used 46 features after dropping IP addresses and port strings. For IoTID20 we used 83 features after removing device-identifier columns.
- **Normalization.** All numerical features were standardized to zero mean and unit variance using statistics computed on the training partition only; the same statistics were applied to validation and test partitions.
- **Missing values.** Features with more than 5% missing values were dropped; remaining missing entries were imputed with the training-partition median.
- **Categorical encoding.** The three datasets contained only numerical features after the above steps; no additional one-hot encoding was required.
- **Train/validation/test split.** Each dataset was split chronologically (to avoid temporal leakage) into 70% training, 10% validation ($D_{\mathrm{val}}$, held server-side), and 20% test. The client data partition was applied to the training split only. The validation and test partitions were never distributed to clients.
- **Client data partitioning.** The training split was distributed to K=10 clients using Dirichlet non-IID sampling with concentration $\alpha_{\mathrm{dir}}=0.5$. Each of the five random seeds controlled both the Dirichlet sampling and the FL training initialization, ensuring full reproducibility of the per-client class distribution.
- **Rare-category definition.** $C_{\mathrm{rare}}$ was fixed before client partitioning according to dataset semantics and under-represented high-risk attack categories, rather than recomputed from each seed by an automatic percentile rule. We used Worms and Shellcode for UNSW-NB15 (0.0663% of all records), seven low-frequency Mirai variants for CICIoT2023 (1.02%), and MITM plus Data Exfiltration for IoTID20 (0.56%). The same rare-category set was used for the AHAG, Recall_rare_ and all cross-dataset sensitivity analyses.
- **Target-client selection.** In all single-client experiments, the target unlearning client was the one whose local dataset contained the highest aggregate proportion of samples belonging to the predefined rare-category set $C_{\mathrm{rare}}$, chosen deterministically after the Dirichlet partition was computed.

#### 3.10.3. Hardware and Software Environment

All experiments were conducted on a server with two NVIDIA A100-SXM4-80GB GPUs (NVIDIA Corporation, Santa Clara, CA, USA), an Intel Xeon Platinum 8358 CPU (Intel Corporation, Santa Clara, CA, USA; 32 cores per socket, 2 sockets), and 512 GB DDR4 RAM. The software stack is as follows: Python 3.10.12, PyTorch 2.1.2 (CUDA 11.8), NumPy 1.24.4, scikit-learn 1.3.2, and the MDPI LATEX template. FL simulation was single-process with sequential client updates per round (no inter-client parallelism during training). The Tab Transformer backbone is implemented in PyTorch using standard nn.MultiheadAttention modules. AHAG gradient computation uses PyTorch autograd with retain_graph=True over the validation batch; each AHAG call requires one forward–backward pass per class in $C_{\mathrm{rare}}\cup \left\{c_{u}\right\}$.

Table 5 provides a detailed computational overhead breakdown to address IoT/edge deployability considerations. AHAG pre-computation is a one-time cost paid before the first unlearning round and need not be repeated across rounds. MRIVA verification adds per-round commitment and comparison overhead that scales with the number of conflict heads and adversary-suspected layers, not with the full model size. The conflict-graph scheduler’s asymptotic cost is $O(|K_{u}{|}^{2}\cdot |H|)$ for graph construction, which is negligible relative to unlearning gradient computations.

**IoT/edge deployment note.** The 8.7 GB peak GPU memory is for server-side unlearning. For client-side local FL training, the measured peak allocated GPU memory is 0.214 GB at a batch size of 64. IoT edge nodes perform ordinary local gradient computation; the server handles the AHAG, DPLCP, and MRIVA centrally. The 256-byte MRIVA per-round commitment is negligible over standard IoT communication links. AHAG overhead scales linearly with the number of rare categories and can be reduced via low-rank QKV approximation [10] for larger transformer backbones.

#### 3.10.4. Random Seeds and Reproducibility

All experiments were repeated five times using seeds {42,123,1234,2024,9999}. Each seed controls: (i) PyTorch global and CUDA random states, (ii) NumPy random state, (iii) Dirichlet client partitioning, and (iv) FL model weight initialization. Tables report mean ± standard deviation (σ) across the five runs; per-run raw values are listed in Appendix A. Additionally, 95% confidence intervals (CI = mean $\pm \;t_{0.025,4}\cdot \sigma /\sqrt{5}$, $t_{0.025,4}=2.776$) are provided for all key metrics in the Supplementary Materials (confidence_intervals_95pct.csv) and are derived directly from the per-seed raw values.

#### 3.10.5. Backbone

Tab Transformer [13]: $L_{T}=6$ transformer layers, H=8 attention heads per layer, $d_{\mathrm{model}}=128$, feed-forward hidden dimension $4d_{\mathrm{model}}=512$, dropout 0.1. The embedding layer maps each of the *M* input features to a $d_{\mathrm{model}}$-dimensional token. The model has approximately 2.1M trainable parameters.

#### 3.10.6. Federated Learning Configuration

K=10 clients; Dirichlet non-IID partitioning ($\alpha_{\mathrm{dir}}=0.5$); $T_{\mathrm{train}}=100$ communication rounds; E=5 local epochs per round; local batch size 64; local SGD learning rate 0.01 with cosine annealing ($\eta_{\min}=10^{-4}$); FedAvg aggregation weighted by local dataset size.

Unlearning hyperparameters: $T_{\mathrm{unl}}=20$ rounds, $\alpha_{\mathrm{base}}=0.01$, $\lambda_{c}=0.8$, $\delta_{\mathrm{crit}}=0.05$, $\delta_{\mathrm{adv}}=0.7$, $\tau_{c}=0.3$, $R_{\mathrm{ref}}=10$ (MRIVA reference trials), Fisher–EWC penalty coefficient $\beta_{\mathrm{EWC}}=400$, Fisher diagonal estimated from 500 samples drawn from $D_{\mathrm{val}}$.

#### 3.10.7. Adversarial Evaluation

Two adversarial settings from recent studies were evaluated: (i) the dormant-backdoor activation scenario of [16], in which malicious clients implant a constrained flow-feature trigger during FL training and exploit the unlearning gradient ascent to activate it, and (ii) the BadUnlearn per-round gradient injection of [17], in which the adversary submits crafted gradient updates at each unlearning round. The flow-feature trigger was applied directly to the tabular network-flow feature vector rather than to any image-domain representation. It perturbed a small fixed subset of valid traffic attributes, including protocol or service indicators, destination-port or service codes, flow duration, packet and byte counts, mean packet length, inter-arrival-time statistics, and packet-direction ratios. Continuous features were clipped to the empirical 1st–99th percentile range of the corresponding training split, categorical features were restricted to values observed in the dataset, and non-negativity as well as duration/byte-count consistency constraints were enforced. Therefore, the ASR and EIR values reported in this paper correspond to a network-flow feature trigger setting for tabular IDS data. Adversarial client fractions are f∈{0.0,0.1,0.2}. For f>0, the malicious clients were selected uniformly at random from $K_{u}$; this selection was fixed across the five seeds to isolate the effect of initialization variance from adversary-selection variance.

#### 3.10.8. Baselines and Their Hyperparameters

Table 6, Table 7 and Table 8 list the hyperparameters used for each baseline. For all methods we used the same Tab Transformer backbone, dataset splits, and FL configuration described above. Where a baseline paper specifies a different backbone or setting, we port the method to our shared setting by following the authors’ publicly available code and applying the same feature set and optimizer.

**Baseline adaptation fairness.** To avoid disadvantaging any published baseline or internal ablation, all methods used the same train/validation/test split, Dirichlet client partition, Tab Transformer backbone, optimizer, unlearning-round budget, random seeds, and evaluation metrics. Where a baseline was originally proposed for a different architecture or data setting, we ported it to the shared Tab Transformer setting using the same feature set and optimizer; no method was given fewer rounds, a smaller validation set, or different data access than ARFU-IDS.
**Baseline and ablation setup.** The comparison includes full retraining, representative federated unlearning baselines, adversarial unlearning defenses, and internal ablation controls. The “Sequential processing” control evaluated in Section 4.3 is an internal scheduler ablation that applies the same single-request unlearning operation to multiple deletion requests serially. This setup separates detection utility, unlearning fidelity, adversarial robustness, and concurrent scheduling efficiency.

#### 3.10.9. Metrics

Detection utility: Macro-F1 (%), ${\mathrm{Recall}}_{\mathrm{rare}}$ (%), FPR (%). Unlearning fidelity: |MIA−50%| (%, lower is better), evaluated using LiRA with 128 shadow models. Adversarial robustness: ASR (↓), Execution Integrity Rate (EIR, ↑); EIR is the fraction of unlearning sessions for which $V_{\mathrm{final}}=\mathrm{PASS}$ and no flow-feature trigger achieves above-chance attack success. Efficiency: $T_{\mathrm{exec}}$ (total online unlearning wall-clock time, seconds, inclusive of MRIVA overhead but excluding the one-time AHAG pre-computation).

## 4. Results

The evaluation is organized to mirror the design requirements defined in Section 2.4. Section 4.1 examines retained detection utility and unlearning fidelity, Section 4.2 evaluates adversarial execution integrity, Section 4.3 studies concurrent multi-client removal, and Section 4.4 analyzes module contributions and threshold sensitivity.

### 4.1. Detection Utility and Unlearning Fidelity

**Figure 6:**
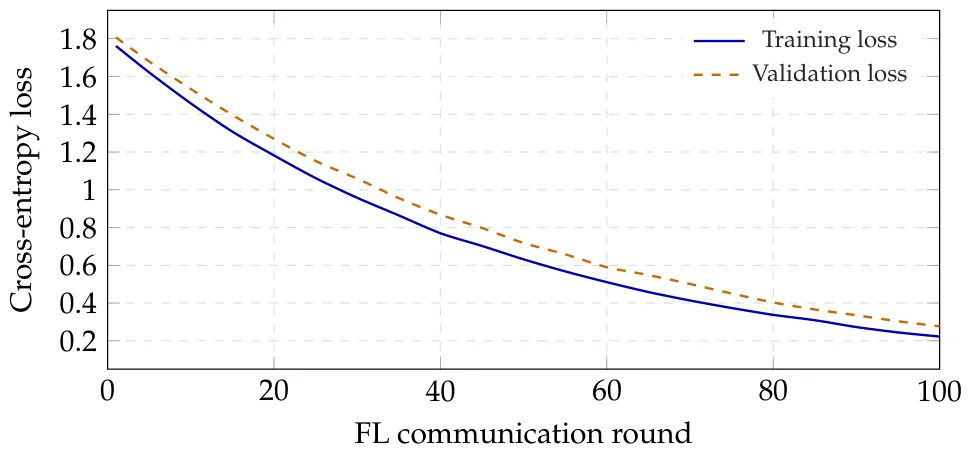
Representative training and validation loss convergence on UNSW-NB15 (ARFU-IDS, seed 42). Both curves decrease steadily and approach convergence by round 100. Per-seed per-round logs for all three datasets are provided in the Supplementary Materials; extended per-seed curves appear in Supplementary Figure S3.

**Figure 7:**
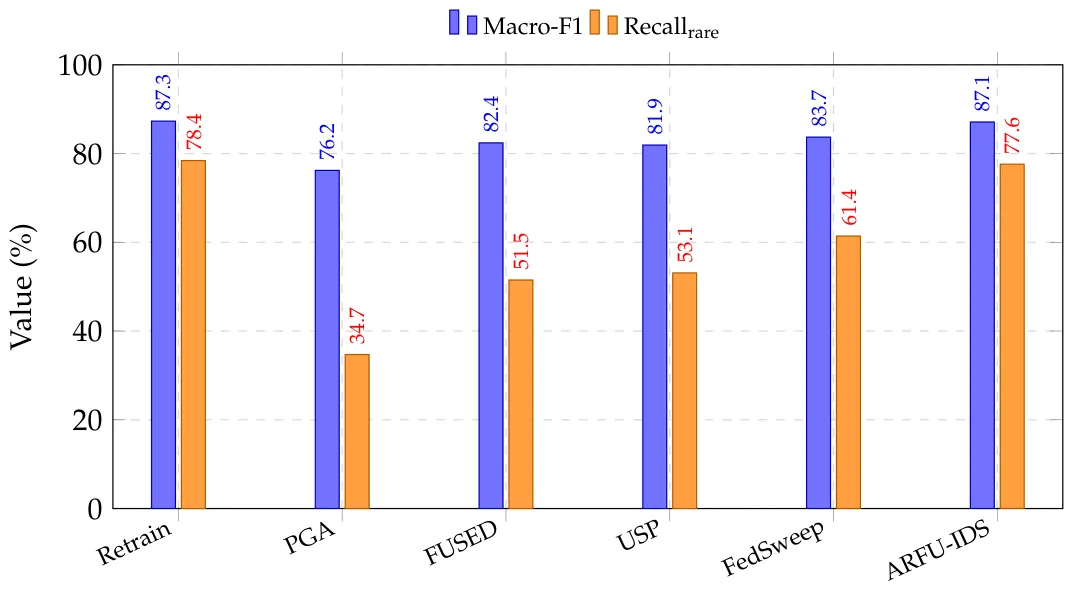
Detection utility on UNSW-NB15, single-client, f=0. Baselines cluster within 10 pp in Macro-F1 but diverge sharply in rare-category recall. ARFU-IDS is the closest non-retraining method to full retraining in ${\mathrm{Recall}}_{\mathrm{rare}}$.

Table 9, Table 10 and Table 11 summarize the cross-dataset results; Table 12 and Table 13 provide the full baseline comparison on UNSW-NB15 with the standard non-adversarial setting (f=0). Figure 6 shows representative training and validation convergence, followed by the method comparison in Figure 7.

ARFU-IDS achieves a Macro-F1 of 87.1%, within 0.2 pp of full retraining. Its ${\mathrm{Recall}}_{\mathrm{rare}}$ of 77.6% represents a 16.2 pp gain over the strongest published non-retraining baseline in this setting (FedSweep, 61.4%). This gap isolates the contribution of transformer-specific head-level attribution: coarse or indiscriminate updates fail to identify the attention-head subspaces encoding rare-category signals, whereas the AHAG preserves these subspaces while removing the target-client contribution. ARFU-IDS achieves an FPR of 3.4%, the lowest among non-retrain methods and only 0.3 pp above retraining’s 3.1%.

On CICIoT2023, ARFU-IDS achieves Macro-F1 84.6% and ${\mathrm{Recall}}_{\mathrm{rare}}$ 71.3% (retrain: 85.1%/72.7%). On IoTID20, ARFU-IDS achieves Macro-F1 91.4% and ${\mathrm{Recall}}_{\mathrm{rare}}$ 86.2% (retrain: 91.8%/87.1%). The smaller gain on IoTID20 is consistent with its lower imbalance ratio, where rare-category information is less concentrated.

Regarding unlearning fidelity, ARFU-IDS achieves an MIA deviation of 2.4%, indicating that the unlearned model remains close to the target retraining behavior while preserving retained detection utility.

Figure 7 plots Macro-F1 and ${\mathrm{Recall}}_{\mathrm{rare}}$ side by side. While baselines remain within a 10 pp band in Macro-F1, they diverge sharply in rare-category recall. ARFU-IDS is the only non-retraining method to remain within 1 pp of full retraining in ${\mathrm{Recall}}_{\mathrm{rare}}$. This property is critical in sensor-network deployments where rare attacks—often the most dangerous—are the hardest to detect.

### 4.2. Adversarial Robustness and Execution Integrity

Table 14 and Table 15 evaluate ASR and EIR, respectively, with escalating adversarial fractions f∈{0.1,0.2} on UNSW-NB15 using the constrained flow-feature trigger defined in Section 3.10. PGA and FUSED remain highly vulnerable because their unlearning updates do not verify the intermediate trajectory. FedSweep and UnlearnGuard reduce ASR partially, but their protection remains incomplete when adversarial clients manipulate per-round updates. ARFU-IDS suppresses ASR to 8.2% at f=0.1 and 9.7% at f=0.2, at least 23.2 pp below the strongest baseline in the table. This improvement is attributable to MRIVA’s trajectory-level verification. EIR decreases modestly from 93.6% to 91.3% as more rounds are steered adversarially, consistent with Proposition 1. The MRIVA verification adds 4.7 s overhead across $T_{\mathrm{unl}}=20$ rounds (approximately 0.24 s per round).

**Figure 8:**
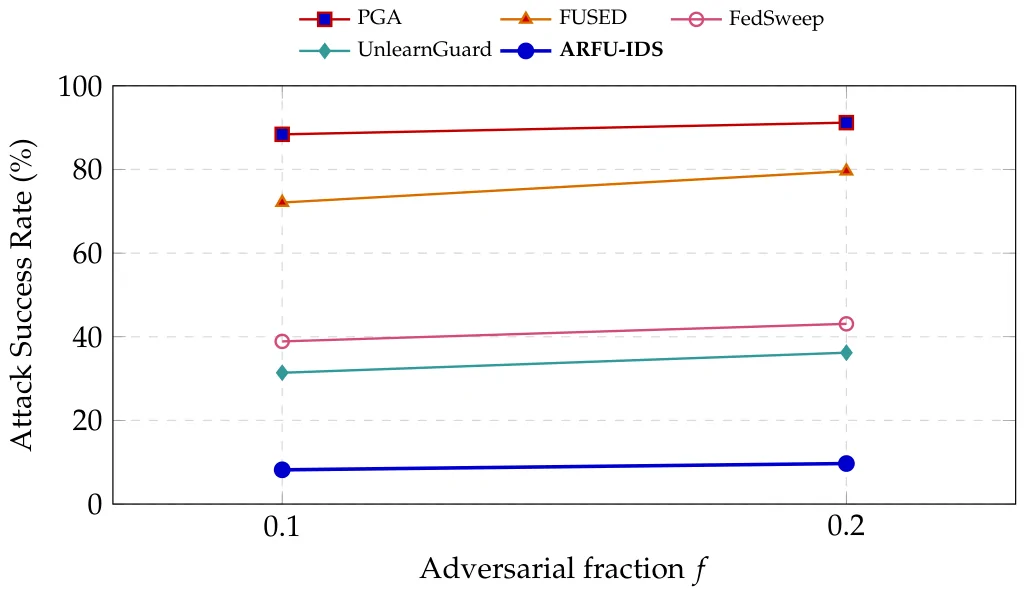
ASR versus adversarial fraction *f* on UNSW-NB15 with constrained flow-feature trigger attacks. ARFU-IDS remains below 10% ASR for both settings, with a 21–83 pp margin over baselines and the flattest degradation trend.

Figure 8 plots ASR as a function of *f*. PGA and FUSED collapse in the BadUnlearn threat model (ASR > 72% in all settings). FedSweep and UnlearnGuard remain above 31% ASR, showing that final-state or partial update filtering is not enough for robust unlearning. ARFU-IDS keeps ASR below 10% for both *f* values and has the flattest degradation trend as the adversarial budget grows.

### 4.3. Concurrent Multi-Client Unlearning

Table 16 reports results for three-client concurrent unlearning on UNSW-NB15 f=0. Naive simultaneous gradient ascent (PGA-Concurrent) collapses ${\mathrm{Recall}}_{\mathrm{rare}}$ to 28.1%, confirming the majority-class dominance problem [9]. Its high MIA deviation (9.4%) reflects global model degradation rather than selective erasure. The sequential-processing control applies the same single-request unlearning operation to client removals one by one, which avoids parallel conflicts but still loses rare-category utility (${\mathrm{Recall}}_{\mathrm{rare}}=64.2\%$) and incurs 83.5 s latency due to serialization.

ARFU-IDS’s conflict-graph scheduling reduces latency to 47.4 s, a 43.3% reduction over the sequential-processing control, while raising ${\mathrm{Recall}}_{\mathrm{rare}}$ to 74.8%, satisfying Condition C2.

**Figure 9:**
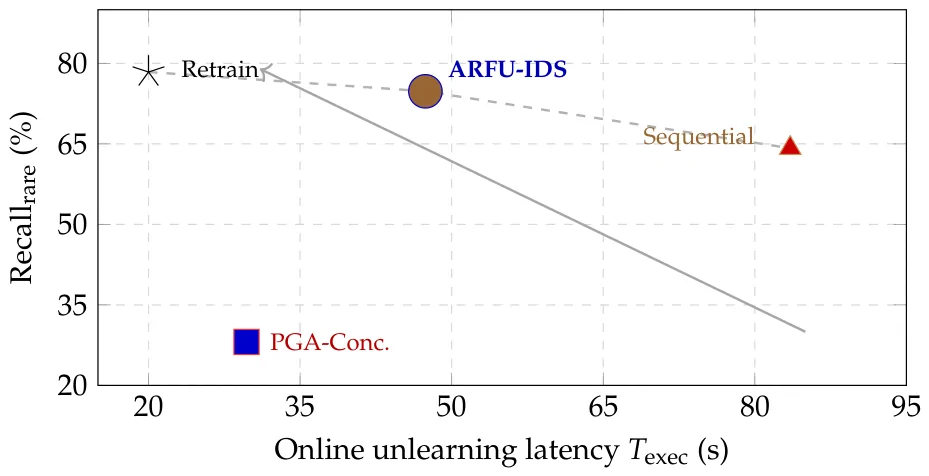
Latency–utility trade-off for concurrent 3-client unlearning on UNSW-NB15 (f=0). ARFU-IDS lies near the Pareto frontier. Dashed segments connect selected high-utility reference solutions.

Figure 9 plots the latency–utility trade-off. ARFU-IDS lies near the Pareto frontier of the evaluated strategies, combining a 43.3% latency reduction over the sequential-processing control with the highest rare-category recall (74.8%) among non-retraining approaches.

### 4.4. Ablation and Sensitivity Analysis

Table 17 presents ablation results on UNSW-NB15 with the Tab Transformer backbone. Module ablations were evaluated at f=0.2; hyperparameter sensitivity rows used f=0.1 to match the grid-search calibration condition for $\delta_{\mathrm{adv}}$.

Removing AHAG drops ${\mathrm{Recall}}_{\mathrm{rare}}$ by 19.4 pp to 58.2% and Macro-F1 by 4.0 pp to 83.1%, confirming head-level conflict resolution as the primary driver of rare-category preservation. The ASR difference between the full system (9.7 ± 0.3%) and the “w/o AHAG” variant (9.1 ± 0.3%) spans overlapping one-sigma intervals; it should not be interpreted as a robustness gain from removing the AHAG. This overlap arises because adversarial robustness at f=0.2 is primarily governed by DPLCP and MRIVA, not by the AHAG; see Table 17.

Removing DPLCP raises ASR from 9.7% to 52.7%, showing that dual-path layer separation is essential for adversarial robustness. Removing MRIVA raises ASR further to 69.4%: DPLCP alone cannot detect trajectory manipulation that constrains per-round deviations within the GCD threshold; MRIVA’s cryptographic commitment closes this gap. Removing the scheduler increases $T_{\mathrm{exec}}$ by 76.5% (from 47.3 s to 83.5 s) with no utility benefit.

**Figure 10:**
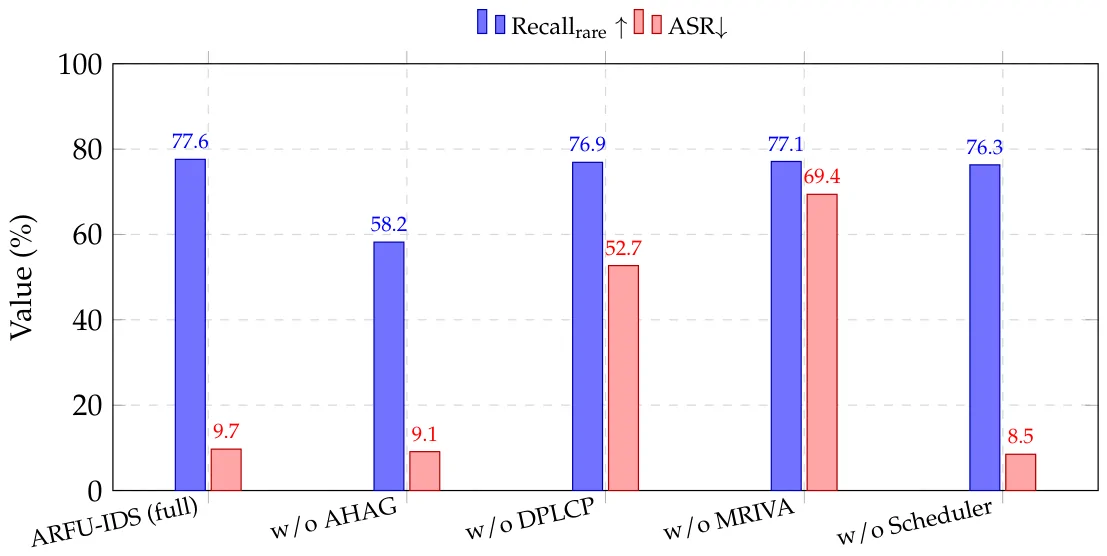
Module-level ablation at f=0.2. In the legend, the upward arrow indicates that higher values are preferable, whereas the downward arrow indicates that lower values are preferable. AHAG drives rare-category utility; DPLCP and MRIVA jointly determine adversarial robustness; the scheduler mainly affects latency.

Figure 10 visualizes the complementary roles of the four modules. AHAG is the dominant driver of rare-category recall. DPLCP and MRIVA jointly dominate adversarial robustness. The scheduler’s contribution is latency rather than accuracy, making it independently adjustable based on deployment constraints.

**Cross-dataset threshold sensitivity.** To address the concern that $\delta_{\mathrm{adv}}$ and the AHAG conflict percentile were calibrated only on UNSW-NB15, Table 18 reports ${\mathrm{Recall}}_{\mathrm{rare}}$ and ASR on CICIoT2023 and IoTID20 when these thresholds are varied. In all cases the default values ($\delta_{\mathrm{adv}}=0.7$, 75th-pct AHAG) remain competitive, and the relative ordering is consistent with Table 17, providing evidence that the calibrated hyperparameters generalize across datasets.

## 5. Discussion

The experimental results support four conclusions.

**AHAG consistently improves rare-category preservation.**Table 9 and Table 12 together with Figure 7 show gains of 19.4, 17.8, and 12.3 pp in ${\mathrm{Recall}}_{\mathrm{rare}}$ on UNSW-NB15, CICIoT2023, and IoTID20, respectively. This pattern confirms that rare-category information is concentrated in specific attention heads and that uniform treatment is ineffective under class imbalance conditions. The smaller gain on IoTID20 is consistent with its lower imbalance ratio.

**DPLCP is essential under adversarially manipulated unlearning conditions.** Removing DPLCP increases ASR to 52.7% at f=0.2, compared with 9.7% for the full system. A task-criticality signal alone is insufficient when adversarial representations reside in layers that appear important to the main task. Because the gradient-consistency discrepancy is attack-agnostic, DPLCP remains applicable even without prior knowledge of the specific flow-feature trigger subset.

**MRIVA closes the trajectory-manipulation gap left by post hoc audits.** Removing MRIVA increases ASR to 69.4%. Monitoring the round-by-round optimization trajectory materially improves robustness at limited additional cost (4.7 s overhead in our setting).

**Conflict-graph scheduling improves concurrent unlearning practicality.** The PGA-Concurrent control achieves lower latency than ARFU-IDS, but it suffers from much lower rare-category recall and substantially higher ASR. ARFU-IDS offers a favorable trade-off for security-critical IoT and WSN deployments where overlapping removal requests must be handled without compromising robustness or minority-attack detection.

**Limitations.** AHAG requires Frobenius-norm gradient evaluation for each attention projection matrix, introducing non-negligible overhead for larger transformers. Gradient checkpointing or low-rank approximation may reduce this cost. Additionally, the current MRIVA threat model does not account for adaptive adversaries with access to $D_{\mathrm{val}}$. Extending verification to this stronger setting is an important future direction.

**Validation set assumptions.** ARFU-IDS follows the server-holds-$D_{\mathrm{val}}$ assumption commonly used in the federated unlearning literature [9]. We acknowledge that in true zero-knowledge FL, a server may hold only a tiny or skewed retained validation set. In that case, AHAG may under-estimate rare-category-critical heads, and MRIVA may produce either overly permissive or overly conservative trajectory decisions. Our experiments used $D_{\mathrm{val}}$ as 10% of the chronological split, which contains the predefined rare categories in proportion to their dataset frequency. For deployments with very small or skewed $D_{\mathrm{val}}$, we recommend stratified public validation slices, bootstrap uncertainty checks on AHAG/DPLCP scores, client-side prototype statistics, conservative MRIVA thresholds, or zero-shot AHAG variants based on public category descriptions; systematic evaluation under these weaker validation assumptions is left for future work.

**Threshold cross-dataset calibration.** The task-critical threshold $\delta_{\mathrm{crit}}=0.05$ was used as a conservative layer-ablation cutoff corresponding to a 5 pp Macro-F1 drop and is fixed across datasets. The adversary-suspicion threshold $\delta_{\mathrm{adv}}=0.7$ and the AHAG conflict percentile were calibrated on UNSW-NB15 and then applied unchanged to CICIoT2023 and IoTID20. Cross-dataset sensitivity results (Table 18) show that the default values maintain competitive Recall_rare_ and ASR on CICIoT2023 and IoTID20, with consistent rank ordering across the tested $\delta_{\mathrm{adv}}$ and AHAG-percentile variants. A systematic sweep of $\delta_{\mathrm{crit}}$ and a more principled leave-one-dataset-out calibration remain worthwhile future work.

## 6. Conclusions

This paper presented ARFU-IDS, a robust federated unlearning framework for transformer-based intrusion detection in IoT and wireless sensor networks. The framework integrates attention-head attribution (AHAG), dual-path layer criticality probing (DPLCP), trajectory-level verification (MRIVA), and conflict-aware scheduling within a single unlearning pipeline.

Experiments on UNSW-NB15, CICIoT2023, and IoTID20 show that ARFU-IDS remains within 0.5 pp of full retraining in Macro-F1 across all three datasets while providing substantially stronger robustness against manipulated unlearning and better concurrent-removal efficiency. On UNSW-NB15, ARFU-IDS achieves 87.1% Macro-F1 and 77.6% ${\mathrm{Recall}}_{\mathrm{rare}}$, reduces ASR to 8.2% at f=0.1 and 9.7% at f=0.2, and shortens concurrent unlearning latency by 43.3% relative to sequentially processing the same deletion requests.

These results suggest that practical FU for transformer-based IDSs should jointly optimize forgetting quality, retained utility, and execution integrity. ARFU-IDS provides a reference framework for sensor-network deployments involving compliance-driven data removal, revocation of compromised clients, and repeated model maintenance under class imbalance conditions. Future work will focus on reducing AHAG overhead for larger transformer backbones, extending the framework to vertical and cross-silo FL settings, supporting multi-category target-client removal, and strengthening trajectory verification under stronger attacker assumptions and formal privacy guarantees [9].

## Figures and Tables

**Table 1:** Coverage of representative federated unlearning methods against the four design requirements. P1: transformer head-level attribution; P2: execution-trajectory integrity; P3: adversary-aware layer separation; P4: concurrent fair scheduling. ✓ = addressed; × = not addressed; P = partially addressed.

Method	P1	P2	P3	P4
PGA [10]	×	×	×	×
FUSED [12]	×	×	×	×
USP [18]	×	×	×	×
UnlearnGuard [17]	×	P	×	×
FedSweep [16]	×	P	P	×
ARFU-IDS (Ours)	✓	✓	✓	✓

**Table 2:** Problem-to-module mapping for ARFU-IDS, Part I: localization, rare-category preservation, and layer treatment.

Design Requirement	Limitation in Existing Methods	ARFU-IDS Response
Transformer-level localization	Coarse neuron-, channel-, or layer-level updates may overlook the attention heads and QKV projection subspaces that encode rare-attack patterns.	AHAG computes QKV Frobenius-gradient scores for each attention head and attenuates updates on heads jointly associated with the target client and rare categories.
Rare-category preservation	Severe class imbalance makes uniform forgetting likely to erase minority-attack representations while removing target-client influence.	AHAG identifies conflict heads and applies differentiated head-level learning rates to preserve rare-category evidence during unlearning.
Adversary-aware layer treatment	Task-critical layers and adversary-influenced layers can be conflated, so a protective constraint may unintentionally preserve malicious representations.	DPLCP combines task-path criticality with gradient consistency discrepancy and assigns protective, aggressive-overwriting, or standard update policies.

**Table 3:** Problem-to-module mapping for ARFU-IDS, Part II: trajectory verification and concurrent-request scheduling.

Design Requirement	Limitation in Existing Methods	ARFU-IDS Response
Trajectory integrity	Final-state audits can miss manipulated intermediate updates or delayed backdoor activation during unlearning.	MRIVA adds round-by-round commitments, reference-gradient deviation checks, and post hoc audit fusion before deployment.
Concurrent deletion requests	Sequential processing increases latency, whereas naive parallel processing may create conflicting removal updates.	The conflict-graph scheduler groups compatible requests for parallel execution and serializes conflicting ones to preserve retained utility.

**Table 4:** Key notation used in ARFU-IDS.

Symbol	Meaning
$\theta^{*}$	Federated model before unlearning.
$\tilde{\theta }$	Model after applying ARFU-IDS unlearning.
$D_{u}$	Target-client data to be forgotten.
$D_{\mathrm{val}}$	Server-held retained validation set.
$K_{u}$	Set of clients submitting unlearning requests.
$h_{l}$	Attention head *h* in transformer layer *l*.
$H_{c}$	Conflict-head set identified by AHAG (≡$H_{\mathrm{conflict}}$).
$L_{\mathrm{task}}$	Task-critical layer set protected by DPLCP.
$L_{\mathrm{adv}}$	Adversary-suspected layer set aggressively overwritten by DPLCP.
$\Delta \theta^{\left(t\right)}$	Projected unlearning update at round *t*.
${\hat{g}}_{t}$	MRIVA reference update direction at round *t*.
$\rho_{t}$	Adaptive MRIVA trajectory-deviation tolerance at round *t*.
$V_{\mathrm{final}}$	Final MRIVA deployment decision.
ASR/EIR	Attack Success Rate/Execution Integrity Rate (%).
$\alpha_{\mathrm{base}}$	Base step size for unlearning gradient updates (default 0.01).
$\lambda_{c}$	Conflict-head gradient-attenuation coefficient in AHAG (default 0.8).
$\eta_{\mathrm{adv}}$	Aggressive overwriting step-size multiplier in DPLCP (default $5\alpha_{\mathrm{base}}$).

**Table 5:** Computational overhead breakdown for ARFU-IDS on UNSW-NB15 (Tab Transformer, 2.1M parameters, K=10, $T_{\mathrm{unl}}=20$). GPU memory is peak resident memory during server-side unlearning. Communication overhead refers to the server-side projection size transmitted per round for MRIVA verification. Aggregate total rows are shown in bold.

Component	Wall Clock	GPU Memory (Peak)	Notes
AHAG pre-computation	3.1 s (one-time)	+0.4 GB	QKV gradients over $D_{\mathrm{val}}$; $O(H\cdot L_{T}\cdot |C_{\mathrm{rare}}|)$ passes
DPLCP per-session	1.8 s (one-time)	+0.2 GB	Layer ablation + GCD over $D_{\mathrm{val}}$
MRIVA per round	0.24 s	+0.03 GB	Hash computation + deviation check; ≤256 bytes commit
MRIVA post hoc (shadow-model audit)	2.6 s (once)	+1.1 GB	128 shadow models; parallelized
Conflict-graph build	<0.01 s (one-time)	negligible	$O(|K_{u}{|}^{2}\cdot H)$
**Total unlearning (single-client)**	**47.3 s**	**8.7 GB**	Server-side ARFU-IDS unlearning
**Total unlearning (3-client conc.)**	**47.4 s**	**9.2 GB**	Parallel phase overlaps

**Table 6:** Hyperparameters used for baseline methods, Part I-a. “Per paper” denotes that the value is taken directly from the respective publication without modification.

Method	Key Hyperparameters
PGA [10]	Projected gradient ascent on $D_{u}$ for $T_{\mathrm{unl}}=20$ rounds; step size 0.01; $\ell_{2}$-projection radius per paper.
FUSED [12]	Sparse adapter dimension r=16; overwriting learning rate 0.05; $T_{\mathrm{unl}}=20$ rounds; other settings per paper.

**Table 7:** Hyperparameters used for baseline methods, Part I-b.

Method	Key Hyperparameters
USP [18]	Top-30% SHAP-selected parameters updated; gradient ascent step 0.01; $T_{\mathrm{unl}}=20$ rounds.
FedSweep [16]	Sweep threshold $\tau_{\mathrm{sweep}}=0.5$; $T_{\mathrm{unl}}=20$ rounds; trigger-aware layer selection per paper.

**Table 8:** Hyperparameters used for baseline methods, Part II. “Per paper” denotes that the value is taken directly from the respective publication without modification.

Method	Key Hyperparameters
UnlearnGuard [17]	Server-side update filtering with cosine-similarity threshold 0.6; $T_{\mathrm{unl}}=20$ rounds; other settings per paper.
Retrain	Full FedAvg retraining on $D\setminus D_{u}$ for 100 rounds with the same FL configuration as the original training.

**Table 9:** Cross-dataset summary of ARFU-IDS and retraining with the standard single-client setting (f=0), Part I: UNSW-NB15. Values are mean ± σ over five runs; per-seed breakdowns are in Appendix A. The corresponding 95% confidence intervals are reported in the Supplementary Materials (confidence_intervals_95pct.csv).

Method	Macro-F1 (%)	Recall _rare_ (%)	Macro-F1 Gap vs. Retrain (pp)
Retrain	87.3 ± 0.2	78.4 ± 0.3	0.0
ARFU-IDS	87.1 ± 0.2	77.6 ± 0.3	−0.2

**Table 10:** Cross-dataset summary of ARFU-IDS and retraining, Part II: CICIoT2023.

Method	Macro-F1 (%)	Recall _rare_ (%)	Macro-F1 Gap vs. Retrain (pp)
Retrain	85.1 ± 0.2	72.7 ± 0.2	0.0
ARFU-IDS	84.6 ± 0.2	71.3 ± 0.3	−0.5

**Table 11:** Cross-dataset summary of ARFU-IDS and retraining, Part III: IoTID20.

Method	Macro-F1 (%)	Recall_rare_ (%)	Macro-F1 Gap vs. Retrain (pp)
Retrain	91.8 ± 0.2	87.1 ± 0.2	0.0
ARFU-IDS	91.4 ± 0.2	86.2 ± 0.2	−0.4

**Table 12:** Detection utility and unlearning fidelity on UNSW-NB15, single-client, f=0, Part I: retraining and generic FU baselines. Values are mean ± σ over five runs. Upward and downward arrows indicate that higher and lower values are preferable, respectively. The corresponding 95% confidence intervals are reported in the Supplementary Materials (confidence_intervals_95pct.csv).

Method	Macro-F1 ↑ (%)	Recall _rare_↑ (%)	FPR ↓ (%)	MIA ↓ (%)
Retrain (gold)	87.3 ± 0.2	78.4 ± 0.3	3.1 ± 0.1	–
PGA [10]	76.2 ± 0.4	34.7 ± 0.6	6.8 ± 0.2	8.9 ± 0.3
FUSED [12]	82.4 ± 0.2	51.5 ± 0.4	5.3 ± 0.2	4.7 ± 0.2
USP [18]	81.9 ± 0.3	53.1 ± 0.5	5.1 ± 0.2	5.2 ± 0.2

**Table 13:** Detection utility and unlearning fidelity on UNSW-NB15, Part II: security-aware baselines and ARFU-IDS. Upward and downward arrows indicate that higher and lower values are preferable, respectively. The best non-retrain result in each column is in **bold**.

Method	Macro-F1 ↑ (%)	Recall _rare_↑ (%)	FPR ↓ (%)	MIA ↓ (%)
FedSweep [16]	83.7 ± 0.2	61.4 ± 0.4	4.9 ± 0.2	3.8 ± 0.2
**ARFU-IDS (TabTrans)**	**87.1 ± 0.2**	**77.6 ± 0.3**	**3.4 ± 0.1**	**2.4 ± 0.2**

**Table 14:** Adversarial robustness on UNSW-NB15 with constrained flow-feature trigger attacks, single-client setting, Part I: ASR. Values are mean ± σ over five runs. The best result in each column is in **bold**.

Method	f=0.1	f=0.2
PGA [10]	88.4 ± 0.4	91.2 ± 0.4
FUSED [12]	72.1 ± 0.5	79.6 ± 0.4
FedSweep [16]	38.9 ± 0.5	43.1 ± 0.5
UnlearnGuard [17]	31.4 ± 0.5	36.2 ± 0.5
**ARFU-IDS (TabTrans)**	**8.2 ± 0.2**	**9.7 ± 0.3**

**Table 15:** Adversarial robustness on UNSW-NB15, Part II: EIR. EIR = 0.0% indicates no trajectory-monitoring capability. The best result in each column is shown in **bold**.

Method	f=0.1	f=0.2
PGA [10]	0.0	0.0
FUSED [12]	0.0	0.0
FedSweep [16]	0.0	0.0
UnlearnGuard [17]	67.3 ± 0.6	63.1 ± 0.6
**ARFU-IDS (TabTrans)**	**93.6 ± 0.3**	**91.3 ± 0.3**

**Table 16:** Concurrent 3-client unlearning on UNSW-NB15, f=0. Values are mean ± σ over five runs. Upward and downward arrows indicate that higher and lower values are preferable, respectively. ΔRecall_rare_ is the absolute drop relative to retraining. The best non-retrain result in each column is in **bold**; for $T_{\mathrm{exec}}$, bold denotes the smallest measured latency even when utility is poor.

Method	Macro-F1 ↑ (%)	Recall_rare_ ↑ (%)	ΔRecall ↓ (%)	MIA ↓ (%)	$T_{\mathrm{exec}}$ ↓ (s)
Retrain (gold)	87.3 ± 0.2	78.4 ± 0.3	0.0	–	–
PGA-Concurrent	71.3 ± 0.5	28.1 ± 0.7	50.3	9.4 ± 0.4	**29.7 ± 0.4**
Sequential processing (no scheduler)	84.9 ± 0.2	64.2 ± 0.4	14.2	2.6 ± 0.2	83.5 ± 0.8
**ARFU-IDS (TabTrans)**	**86.9 ± 0.2**	**74.8 ± 0.4**	**3.6**	**2.3 ± 0.1**	47.4 ± 0.6

**Table 17:** Ablation and sensitivity analysis on UNSW-NB15 (Tab Transformer). Values are mean ± σ over five runs. Upward and downward arrows indicate that higher and lower values are preferable, respectively. Module ablations at f=0.2; hyperparameter rows at f=0.1. The ASR difference between the full system (9.7 ± 0.3%) and the “w/o AHAG” variant (9.1 ± 0.3%) spans overlapping one-sigma intervals and should not be interpreted as a robustness gain from removing the AHAG. Reference configurations are in **bold**. The corresponding 95% confidence intervals are reported in the Supplementary Materials (confidence_intervals_95pct.csv).

Variant	Macro-F1 (%)	Recall _rare_ (%)	ASR ↓ (%)	EIR ↑ (%)	$T_{\mathrm{exec}}$ (s)
Module ablations (f=0.2)					
**ARFU-IDS (full)**	**87.1 ± 0.2**	**77.6 ± 0.3**	**9.7 ± 0.3**	**91.3 ± 0.3**	**47.3 ± 0.6**
w/o AHAG (Unif. Ascent)	83.1 ± 0.3	58.2 ± 0.5	9.1 ± 0.3	93.1 ± 0.5	44.8 ± 0.5
w/o DPLCP	86.8 ± 0.2	76.9 ± 0.3	52.7 ± 0.8	92.8 ± 0.5	46.1 ± 0.5
w/o MRIVA	86.9 ± 0.2	77.1 ± 0.3	69.4 ± 1.0	–	42.6 ± 0.5
w/o Scheduler (seq.)	86.7 ± 0.2	76.3 ± 0.3	8.5 ± 0.3	93.4 ± 0.5	83.5 ± 1.1
Hyperparameter sensitivity (f=0.1)					
AHAG @ 50th pct	87.0 ± 0.2	76.3 ± 0.3	8.4 ± 0.3	93.5 ± 0.4	47.1 ± 0.6
**AHAG @ 75th pct (def.)**	**87.1 ± 0.2**	**77.6 ± 0.3**	**8.2 ± 0.3**	**93.6 ± 0.4**	**47.3 ± 0.6**
AHAG @ 90th pct	87.0 ± 0.2	77.9 ± 0.3	9.3 ± 0.3	93.2 ± 0.4	47.6 ± 0.6
Hyperparameter sensitivity (continued; f=0.1)					
$\delta_{\mathrm{adv}}=0.5$	86.3 ± 0.3	76.8 ± 0.3	9.8 ± 0.3	94.1 ± 0.4	47.5 ± 0.6
**$\delta_{\mathrm{adv}}=0.7$ (def.)**	**87.1 ± 0.2**	**77.6 ± 0.3**	**8.2 ± 0.3**	**93.6 ± 0.4**	**47.3 ± 0.6**
$\delta_{\mathrm{adv}}=0.9$	86.9 ± 0.2	77.4 ± 0.3	14.6 ± 0.4	93.3 ± 0.4	47.2 ± 0.6

**Table 18:** Cross-dataset threshold sensitivity (f=0.1, single-client). Values are mean ± σ over five runs. Upward and downward arrows indicate that higher and lower values are preferable, respectively. Bold denotes the default configuration. The corresponding 95% confidence intervals are reported in the Supplementary Materials (confidence_intervals_95pct.csv).

Variant	CICIoT2023		IoTID20	
	Recall_rare_ ↑ (%)	ASR ↓ (%)	Recall_rare_ ↑ (%)	ASR ↓ (%)
$\delta_{\mathrm{adv}}=0.5$	69.6 ± 0.5	11.3 ± 0.2	85.6 ± 0.2	10.4 ± 0.4
$\delta_{\mathrm{adv}}=0.7$ **(def.)**	**71.2 ± 0.3**	**8.9 ± 0.4**	**86.2 ± 0.4**	**8.9 ± 0.4**
$\delta_{\mathrm{adv}}=0.9$	70.8 ± 0.3	15.2 ± 0.4	85.7 ± 0.4	14.8 ± 0.2
AHAG @ 50th pct	69.7 ± 0.3	9.1 ± 0.2	85.0 ± 0.3	9.0 ± 0.3
**AHAG @ 75th pct (def.)**	**71.2 ± 0.1**	**9.1 ± 0.3**	**86.2 ± 0.4**	**8.8 ± 0.5**
AHAG @ 90th pct	71.3 ± 0.3	10.6 ± 0.8	86.3 ± 0.4	10.1 ± 0.3

## Data Availability

The three benchmark datasets are publicly available: UNSW-NB15 (https://research.unsw.edu.au/projects/unsw-nb15-dataset (accessed on 20 July 2026)), CICIoT2023 (https://www.unb.ca/cic/datasets/iotdataset-2023.html (accessed on 20 July 2026)), and IoTID20 via its public dataset repository. The implementation and reproducibility artifacts are available in the accompanying anonymized GitHub repository. The Supplementary Materials contain configuration files, preprocessing utilities and class mappings, split and client-partition metadata, random-seed utilities, raw per-seed results, per-round logs, an environment file, and reference command templates.

## Supplementary Materials

The following supporting information can be downloaded at: https://www.mdpi.com/article/10.3390/s26185911/s1, Figure S1: AHAG Attention-Head Attribution (UNSW-NB15, seed 42) (file: Figure_S1_Attention_Head_Attribution_Heatmaps.pdf); Figure S2: Embedding projections before and after unlearning (file: Figure_S2_Embedding_Projections_Before_and_After_Unlearning.pdf); Figure S3: Training and validation loss convergence curves (file: Figure_S3_Training_and_Validation_Loss_Convergence_Curves.pdf); Table S1: Supplementary statistical summary containing the 95% confidence intervals for the key metrics reported in the manuscript (file: confidence_intervals_95pct.csv); Data S1: Reproducibility materials (folder: Data_S1_Reproducibility_Materials).

## Appendix A. Per-Run Raw Results

This appendix reports the raw metric values from each of the five independent runs used to compute the means and standard deviations in the main tables. All runs were executed sequentially on the same hardware (two NVIDIA A100-SXM4-80 GB GPUs) in the configuration described in Section 3.10. The Supplementary Materials includes per-round metric logs and wall-clock timing fields; detailed system-level GPU-utilization traces are available from the corresponding author upon reasonable request.

### Appendix A.1. ARFU-IDS on UNSW-NB15: Detection Utility (f=0, Single-Client)

**Table A1:** Per-seed detection utility results for ARFU-IDS on UNSW-NB15 (f=0, single-client). The “Mean” and “σ” rows match Table 12. Mean rows are shown in bold.

Seed	Macro-F1 (%)	Recall_rare_ (%)	FPR (%)	MIA (%)
42	87.3	77.9	3.3	2.3
123	86.9	77.2	3.5	2.6
1234	87.1	77.7	3.4	2.4
2024	87.2	77.4	3.4	2.2
9999	87.0	77.8	3.4	2.5
**Mean**	**87.1**	**77.6**	**3.4**	**2.4**
σ	0.16	0.29	0.07	0.16

### Appendix A.2. ARFU-IDS on UNSW-NB15: Adversarial Robustness

**Table A2:** Per-seed adversarial robustness results for ARFU-IDS on UNSW-NB15. Means and standard deviations match Table 14. Mean rows are shown in bold.

Seed	ASR (%), f=0.1	EIR (%), f=0.1	ASR (%), f=0.2	EIR (%), f=0.2
42	8.0	94.0	9.5	91.7
123	8.5	93.2	10.1	90.8
1234	8.1	93.8	9.6	91.4
2024	8.4	93.4	9.8	91.2
9999	8.0	93.6	9.5	91.4
**Mean**	**8.2**	**93.6**	**9.7**	**91.3**
σ	0.24	0.32	0.26	0.33

### Appendix A.3. ARFU-IDS on UNSW-NB15: Concurrent Unlearning (f=0, 3 Clients)

**Table A3:** Per-seed concurrent unlearning results for ARFU-IDS on UNSW-NB15. Means and standard deviations match Table 16. Mean rows are shown in bold.

Seed	Macro-F1 (%)	Recall_rare_ (%)	MIA (%)	$T_{\mathrm{exec}}$ (s)
42	87.0	75.1	2.2	46.9
123	86.7	74.3	2.5	48.1
1234	87.0	74.9	2.3	47.7
2024	87.1	75.2	2.2	46.6
9999	86.7	74.5	2.3	47.5
**Mean**	**86.9**	**74.8**	**2.3**	**47.4**
σ	0.19	0.39	0.12	0.61

### Appendix A.4. Cross-Dataset Per-Seed Summary (ARFU-IDS, f=0, Single-Client)

**Table A4:** Per-seed Macro-F1 and ${\mathrm{Recall}}_{\mathrm{rare}}$ for ARFU-IDS on CICIoT2023 and IoTID20. Means match Table 9. Mean rows are shown in bold.

Seed	CICIoT2023		IoTID20	
	Macro-F1 (%)	Recall_rare_ (%)	Macro-F1 (%)	Recall_rare_ (%)
42	84.8	71.6	91.5	86.4
123	84.3	70.9	91.2	85.9
1234	84.6	71.4	91.4	86.3
2024	84.7	71.2	91.6	86.4
9999	84.6	71.4	91.3	86.0
**Mean**	**84.6**	**71.3**	**91.4**	**86.2**
σ	0.19	0.27	0.16	0.24

All five seeds consistently reproduce the rank ordering of methods reported in the main tables. The observed standard deviations are all below 1.1 pp for utility metrics, below 1.0 pp for ASR/EIR, and below 1.2 s for $T_{\mathrm{exec}}$, confirming stable convergence across random initializations and client partitions.
