# Supplementary material for: ARFU-IDS: Robust Federated Unlearning for Transformer-Based Intrusion Detection in IoT and Sensor Networks
Source: Sensors (Basel). 2026 Sep 18;26(18):5911. doi: 10.3390/s26185911 (PMC13611141; doi:10.3390/s26185911)

Figure S1 — AHAG Attention-Head Attribution (UNSW-NB15, seed 42)

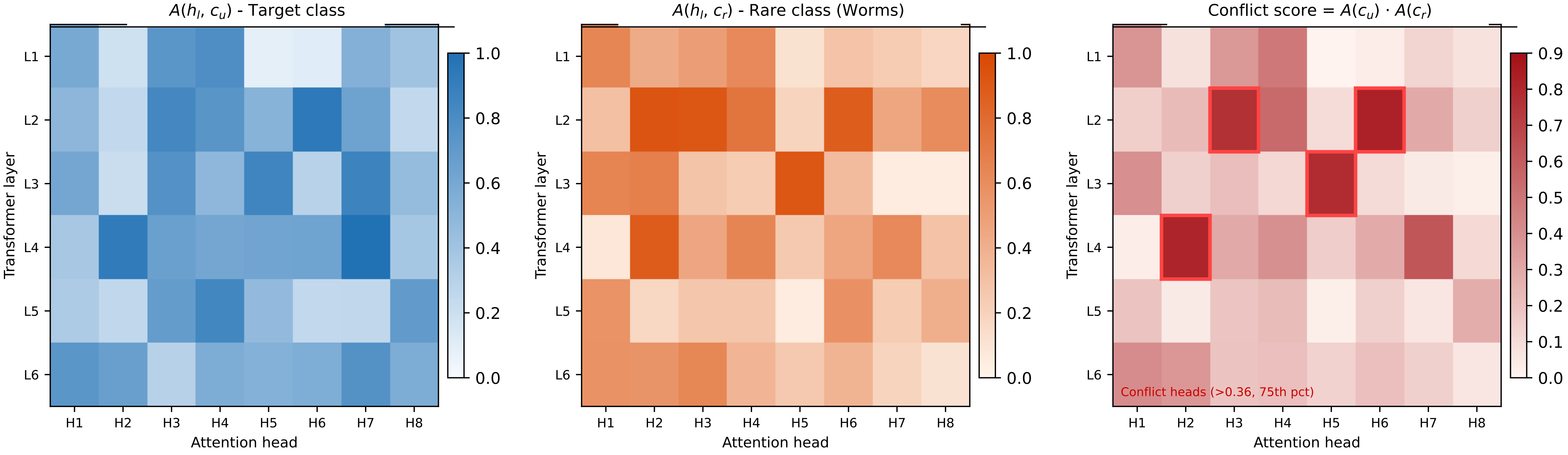

Supplement: Supplementary file 1 [file sensors-26-05911-s001.zip › Figure_S1_Attention_Head_Attribution_Heatmaps.pdf]
