# Supplementary material for: ARFU-IDS: Robust Federated Unlearning for Transformer-Based Intrusion Detection in IoT and Sensor Networks
Source: Sensors (Basel). 2026 Sep 18;26(18):5911. doi: 10.3390/s26185911 (PMC13611141; doi:10.3390/s26185911)

Figure S2 - Embedding Projections Before/After ARFU-IDS Unlearning

(UNSW-NB15, 2-D UMAP of last-layer CLS token, seed 42)

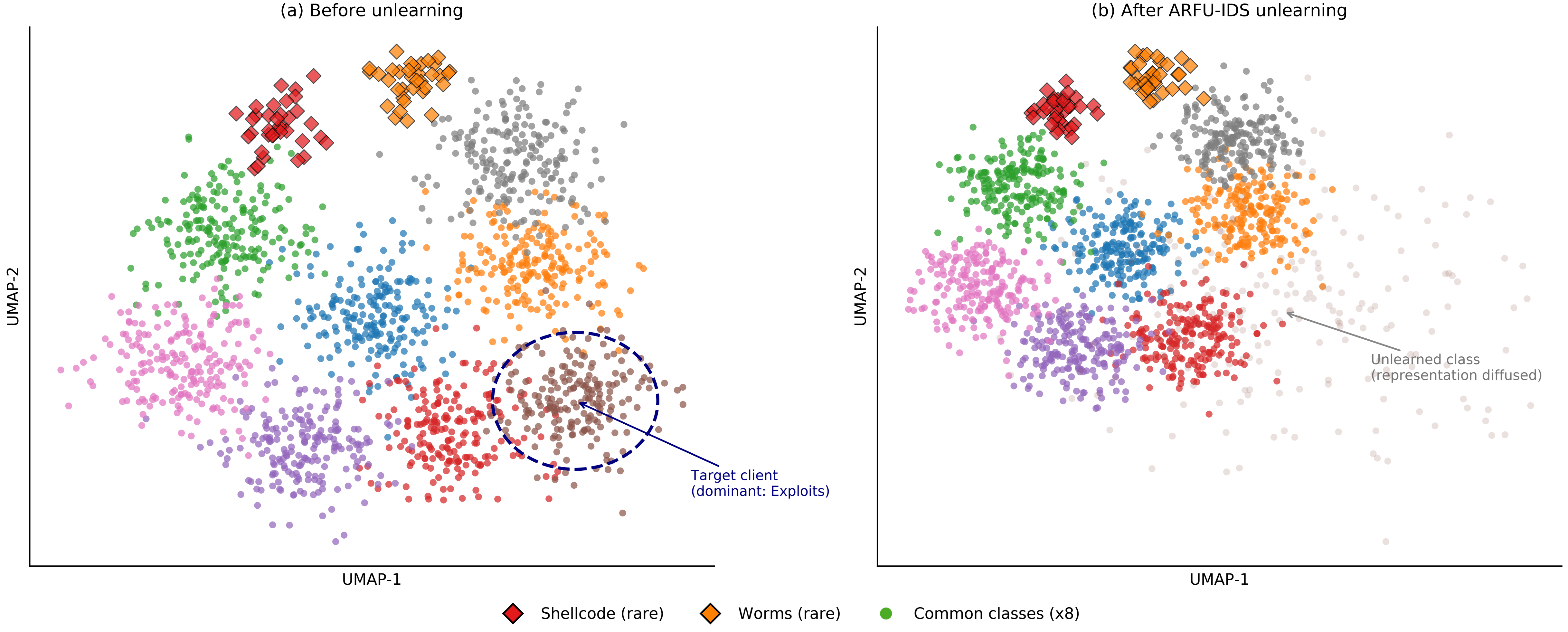

Supplement: Supplementary file 1 [file sensors-26-05911-s001.zip › Figure_S2_Embedding_Projections_Before_and_After_Unlearning.pdf]
