# Supplementary material for: ARFU-IDS: Robust Federated Unlearning for Transformer-Based Intrusion Detection in IoT and Sensor Networks
Source: Sensors (Basel). 2026 Sep 18;26(18):5911. doi: 10.3390/s26185911 (PMC13611141; doi:10.3390/s26185911)
Supplement: Supplementary file 1 [file sensors-26-05911-s001.zip › Figure_S3_Training_and_Validation_Loss_Convergence_Curves.pdf]

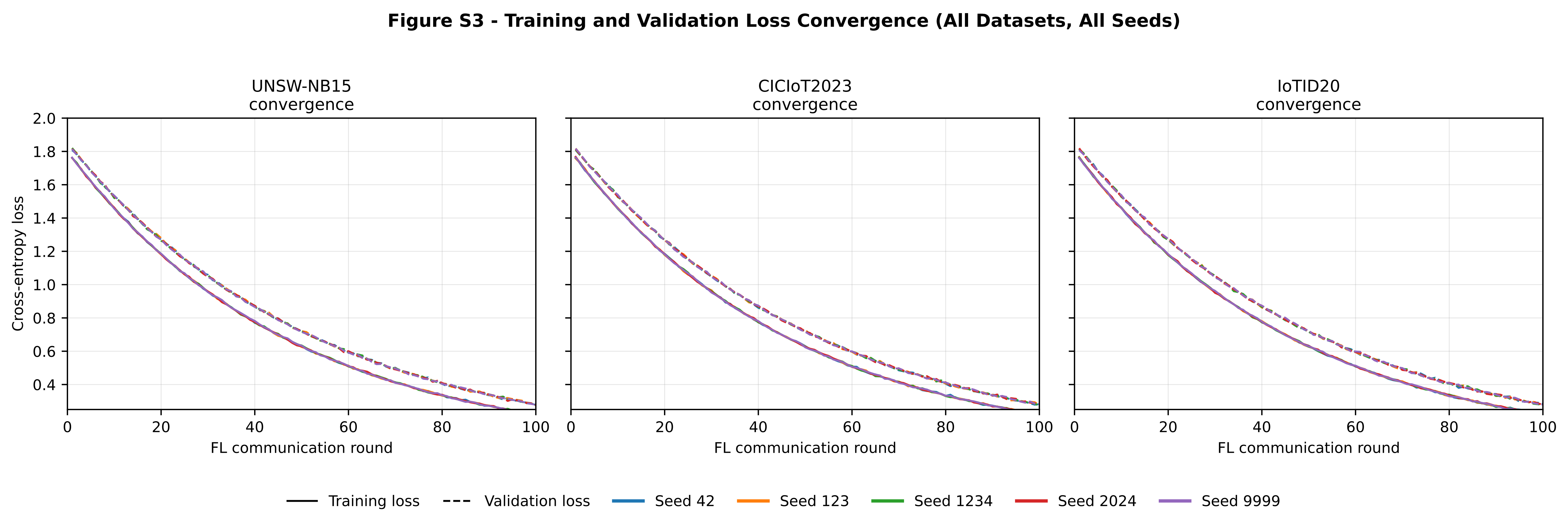

Figure S3 - Training and Validation Loss Convergence (All Datasets, All Seeds)
UNSW-NB15
convergence
CICIoT2023
convergence
IoTID20
convergence
Cross-entropy loss
2.0
1.8
1.6
1.4
1.2
1.0
0.8
0.6
0.4
0
20
40
60
80
100
FL communication round
Training loss
Validation loss
Seed 42
Seed 123
Seed 1234
Seed 2024
Seed 9999
